# The multifaceted role of c-di-AMP signaling in the regulation of *Porphyromonas gingivalis* lipopolysaccharide structure and function

**DOI:** 10.3389/fcimb.2024.1418651

**Published:** 2024-06-12

**Authors:** Shirin Ghods, Artur Muszyński, Hyojik Yang, Ratnam S. Seelan, Asal Mohammadi, Jacob S. Hilson, Griffin Keiser, Frank C. Nichols, Parastoo Azadi, Robert K. Ernst, Fata Moradali

**Affiliations:** ^1^ Department of Oral Immunology and Infectious Diseases, School of Dentistry, University of Louisville, Louisville, KY, United States; ^2^ Complex Carbohydrate Research Center, University of Georgia, Athens, GA, United States; ^3^ Department of Microbial Pathogenesis, School of Dentistry, University of Maryland, Baltimore, MD, United States; ^4^ Division of Periodontology, University of Connecticut School of Dental Medicine, Farmington, CT, United States

**Keywords:** *Porphyromonas gingivalis*, lipopolysaccharide, structural variations, C-di-AMP signaling, cellular bioenergetics

## Abstract

**Background:**

This study unveils the intricate functional association between cyclic di-3’,5’-adenylic acid (c-di-AMP) signaling, cellular bioenergetics, and the regulation of lipopolysaccharide (LPS) profile in *Porphyromonas gingivalis*, a Gram-negative obligate anaerobe considered as a keystone pathogen involved in the pathogenesis of chronic periodontitis. Previous research has identified variations in *P. gingivalis* LPS profile as a major virulence factor, yet the underlying mechanism of its modulation has remained elusive.

**Methods:**

We employed a comprehensive methodological approach, combining two mutants exhibiting varying levels of c-di-AMP compared to the wild type, alongside an optimized analytical methodology that combines conventional mass spectrometry techniques with a novel approach known as FLAT^n^.

**Results:**

We demonstrate that c-di-AMP acts as a metabolic nexus, connecting bioenergetic status to nuanced shifts in fatty acid and glycosyl profiles within *P. gingivalis* LPS. Notably, the predicted regulator gene *cdaR*, serving as a potent regulator of c-di-AMP synthesis, was found essential for producing N-acetylgalactosamine and an unidentified glycolipid class associated with the LPS profile.

**Conclusion:**

The multifaceted roles of c-di-AMP in bacterial physiology are underscored, emphasizing its significance in orchestrating adaptive responses to stimuli. Furthermore, our findings illuminate the significance of LPS variations and c-di-AMP signaling in determining the biological activities and immunostimulatory potential of *P. gingivalis* LPS, promoting a pathoadaptive strategy. The study expands the understanding of c-di-AMP pathways in Gram-negative species, laying a foundation for future investigations into the mechanisms governing variations in LPS structure at the molecular level and their implications for host-pathogen interactions.

## 1 Introduction

Periodontitis in adults represents a widespread infection and inflammation affecting the periodontal tissues supporting the teeth and is associated with the onset of various inflammatory diseases (Hajishengallis and Lamont, 2012; Lamont et al., 2018). Current etiological models propose that periodontitis is induced by a consortium of virulent bacteria, predominantly Gram-negative bacteria persisting below the gum line. Among these, *Porphyromonas gingivalis*, a Gram-negative anaerobe belonging to Bacteroidota, is recognized as a pivotal species in periodontitis progression. *P. gingivalis* produces various virulence factors, notably lipopolysaccharides (LPS), generally known as endotoxins. The presence of *P. gingivalis* LPS in the bloodstream of periodontitis patients underscores its potential to dysregulate immune functions in periodontal tissues and distal organs (Deleon-Pennell et al., 2013, 2014; Napa et al., 2017). Despite this, the pathoadaptive mechanisms enabling *P. gingivalis* to thrive in the host environment and establish persistent infections remain inadequately understood. Accumulated evidence suggests that *P. gingivalis* can adapt to its host environment and promote persistent infections by modifying the composition and immunostimulatory characteristics of LPS in response to biologically relevant stimuli, particularly in the presence of hemin - an essential iron source for *P. gingivalis* proliferation (Al-Qutub et al., 2006; Coats et al., 2009; Guo et al., 2010; Hajishengallis et al., 2012; Olsen et al., 2017; Madej et al., 2021). In addition to variations in the LPS profile, inconsistencies in the selected approaches to study the LPS profile and its subsequent effect on function have contributed to disparities among previous findings. Some studies have reported highly unusual and differential immune and host responses to *P. gingivalis* LPS (Millar et al., 1986; Bainbridge and Darveau, 2001; Darveau et al., 2002; Dixon and Darveau, 2005). Consequently, our understanding of the mechanistic basis of the regulation of *P. gingivalis* LPS structure and function and its subsequent contribution to disease progression has remained limited.

In general, LPS macromolecules exhibit tripartite structures, comprising lipid A, core oligosaccharide, and outer polysaccharide. Numerous studies have enhanced our understanding of lipid A components in *P. gingivalis* LPS, revealing at least four major lipid A species with varying degrees of acylation/phosphorylation and immunostimulatory potential (Simpson et al., 2000; Al-Qutub et al., 2006; Coats et al., 2009). Additionally, two forms of LPS, O- and A-LPS, composed of distinct polysaccharide components, have been reported in *P. gingivalis* (Rangarajan et al., 2008; Swietnicki and Caspi, 2021). Our previous research has provided some initial insights into the modulation of *P. gingivalis* LPS profile through cyclic di-3’, 5’-adenylic acid (c-di-AMP) second messenger signaling (Moradali et al., 2022). We observed that shifts in the cellular c-di-AMP pool change the integrity and homeostasis of the cell envelope, immunoreactivity of LPS, susceptibility of the species to antimicrobials, and virulence potential (Moradali et al., 2022). C-di-AMP signaling, a well-established regulatory mechanism in Gram-positive species, plays a crucial role in bacterial pathoadaptation and the establishment of chronic infections (Foreman-Wykert and Miller, 2003; Choi and Groisman, 2013; Pontes et al., 2015; Moradali et al., 2017; Olsen and Singhrao, 2018). However, its mechanistic basis in Gram-negative bacteria, particularly in asaccharolytic anaerobes, and its contribution to pathogenesis, have remained largely unknown. Our recent findings demonstrated that c-di-AMP signaling is indispensable for *P. gingivalis* physiology and growth, influencing its survival and virulence potential (Moradali et al., 2022). We identified key components of the c-di-AMP signaling pathway in *P. gingivalis*, including the c-di-AMP synthase gene (*dac_pg_*; PGN_0523), the c-di-AMP phosphodiesterase gene (*pde_pg_*; PGN_0521), and the predicted regulatory gene *cdaR* (PGN_1486), using single-gene knockout mutants and strains complemented with relevant genes (Moradali et al., 2022). Complementation of the mutants with *pde_pg_* or *cdaR* restored c-di-AMP synthesis to WT levels, ruling out suppressive mutation or polar effects. We also showed that *pde_pg_* in *P. gingivalis* acts as an atypical c-di-AMP phosphodiesterase and positively regulates c-di-AMP synthesis and impedes a decrease in c-di-AMP concentration despite encoding conserved amino acid motifs for phosphodiesterase activity (Moradali et al., 2022). All these genes are required for the incorporation of ATP into c-di-AMP, particularly in the presence of alternate energy sources. In the presence of exogenous pyruvate, an important alternative carbon and energy source, the increased availability of ATP alters the metabolic state of the slow-growing and asaccharolytic anaerobe toward enhanced gluconeogenesis and an altered bioenergetic state (Moradali and Davey, 2021).

In this study, we show that shifts in cellular c-di-AMP concentration have a significant effect on the regulation of LPS profiles in *P. gingivalis*. We also explore a critical role of c-di-AMP signaling in linking bioenergetic status and the regulation of LPS profile, which subsequently impacts its biological activity and immunostimulatory potential. Our research, employing wild type (WT) strain 381 and its derivative mutants Δ*pde_pg_* and Δ*cdaR* with varying c-di-AMP levels, combines conventional and innovative methodologies to shed light on the critical role of c-di-AMP signaling system in the physiology and pathogenesis of *P. gingivalis*, and potentially other members of Bacteroidota. Since c-di-AMP signaling is absent in mammalian cells and crucial for *P. gingivalis* survival, it stands as a unique target for therapeutic development against periodontal pathogens.

## 2 Materials and methods

### 2.1 Bacterial strains and culture conditions

*P. gingivalis* (*Pg*) wild-type strain 381 and mutant strains, 381 Δ*pde_pg_* (*pde_pg_*; PGN_0521) and 381 Δ*cdaR* (*dac_pg_*; PGN_0523) (Moradali et al., 2022) were used in the present study. Using frozen stocks as the initial inoculum, these strains were grown on Trypticase Soy Agar plates supplemented with 5 µg/ml hemin, 1 µg/ml menadione, and 5% defibrinated sheep blood (Northeast Laboratory Services, USA), and incubated at 37°C in an anaerobic chamber (Coy Lab Products) containing 5% hydrogen, 10% carbon dioxide, and 85% nitrogen for 3 days. All procedures described in this section were performed in the anaerobic chamber. Subsequently, *Pg* was cultivated in 10 ml of pre-reduced BD™ Bacto™ Tryptic Soy Broth without Dextrose (TSB) supplemented with 0.5% Yeast extract (Fisher BioReagents™), 1 µg/ml hemin and 1 µg/ml menadione (TSBYK) at 37°C overnight. The above culture was diluted in a 10 ml of fresh medium to an optical density (OD_600_) of 0.2 and allowed to grow anaerobically for about 16 hours. For biofilm analysis, 100 µl of the cells were plated on a TSBYK agar plate supplemented with 1 or 10 µg/ml hemin and incubated for 24 h at 37°C; the remaining culture was incubated for an additional 24 h at 37°C for isolation of planktonic cells.

### 2.2 ELISA quantification of c-di-AMP and ATP

Metabolites were extracted according to previously published protocols (Moradali et al., 2022). C-di-AMP levels were quantified using a c-di-AMP Enzyme-Linked Immunosorbent Assay (ELISA) Kit (Cayman Chemical, USA), according to the manufacturer’s protocol. Briefly, cells were cooled on ice for 20 min and subsequently harvested by centrifugation at 4700 × g, 4°C for 10 min. The collected cells were washed and then suspended in 300 μl of ice-cold extraction solvent composed of acetonitrile/methanol/water (2/2/1, v/v/v). The suspension underwent an additional 15-min incubation on ice, followed by heating at 95°C for 10 min and subsequent cooling on ice. After centrifugation at 20,800 × g, 4°C for 10 min, the supernatants were gathered, and the pellets were subjected to two additional extractions with solvent without heating. The combined supernatants from the three extraction steps were stored at −20°C overnight for protein precipitation. After centrifugation the pellets were collected for protein quantification using the BCA Kit (Thermo Scientific). The supernatants were dried using a Speed-Vac (Thermo Scientific) at 40°C. The dried samples were reconstituted in 200 μl of sample solvent and vortexed for 30 sec. Fifty µl of the sample was added to each well, followed by the addition of 50 µl of c-di-AMP-HRP tracer from the kit (Item No. 401960). Within a 15-min window, 50 µl of the antibody was introduced. The plate was covered and incubated on an orbital shaker at room temperature (RT) for 2 h. After five washes with the Wash Buffer provided in the kit, 175 µl of 3,3’,5,5’-tetramethylbenzidine (TMB) substrate solution (Item No. 400074) from the c-di-AMP ELISA Kit was added. Upon adding 75 µl of the HRP Stop Solution from the kit, the reaction was terminated, and the plate was read at 450 nm using SpectraMax iD3 (Molecular Devices, USA). A standard plot based on % Bound/Maximum Bound (%B/B0) against serial c-di-AMP dilutions was created using a four-parameter logistic fit (**Supplementary Figure 11**). The %B/B0 value for each sample was determined, and the c-di-AMP concentration was determined through a standard curve.

The ATPlite™ Luminescence Assay System (https://www.perkinelmer.com) was used to quantify ATP levels in the metabolic extracts, isolated as described above. Briefly, 50 µl of each sample was added in triplicate to microtiter plate wells. Subsequently, 50 µl of the kit’s lysis buffer and 60 µl of its reconstituting buffer were added, mixed, and agitated for 5 min at 700 rpm on a rotational shaker. Following this, 50 µl of the reconstituted enzyme was added to the wells, mixed for 3 min, and luminescence was immediately measured at 578 nm using SpectraMax iD3 (Molecular Devices). The ATP concentration of each sample was calculated based on a standard curve established in the same plate utilizing the kit’s ATP standards (**Supplementary Figure 11**).

### 2.3 LPS isolation

The cell pellets, prepared as described above, were suspended in deionized water, and pre-warmed to 68°C. LPS extraction was carried out following the procedure of Westphal and Jann (Westphal and Jann, 1965). Briefly, an equal volume of phenol was added to the cell suspension and extraction was carried out for 20 min at 68°C, with gentle stirring. The sample was then cooled in an ice bath and centrifuged for 20 min at 10,000 x g at 4°C. The aqueous upper phase was collected, and the remaining phenol phase was extracted twice more with water. The combined aqueous phases and the phenol phase were dialyzed (12–14 kDa MWCO) against several changes of water, for about 4 days. The dialyzed samples were then freeze-dried. The crude LPS sample was dissolved to a final concentration of 10 mg/ml in a sterile buffer (50 mM MgCl_2_.6H_2_O and 20 mM NaOAc.3H_2_O; pH7.6) and the nucleic acids were digested with ~500 U of Benzonase (Sigma-Aldrich) for 16 h at 37°C with gentle agitation. The proteins were digested with Proteinase K (7.5 U/ml of LPS solution) and incubated with gentle agitation for 1 h at 50°C, followed by overnight incubation for 16 h at 37°C. The buffer and resulting nucleotides and peptides were excluded by dialysis (12–14 kDa MWCO) against several exchanges of water at 4°C and centrifugation at 100,000 x g for 6 h at 4°C. The ultracentrifugation pellets containing clean LPS were suspended in water, freeze-dried, and subjected to analysis. It is important to note that once purified, we use a single batch of LPS across various analytical experiments, as well as *in vitro* and *in vivo* assays, to establish correlations between different analytical methods and between compositional data and biological activities.

### 2.4 FLAT^n^ technique

A Bruker Matrix-Assisted Laser Desorption/Ionization trapped ion mobility spectrometry Time-of-Flight Mass Spectrometry (MALDI (tims TOF) MS) was performed as previously described (Yang et al., 2022, 2023). Briefly, a MALDI (tims TOF) MS was used for FLAT (direct biomass MS for visualization of lipid A primary molecular ions), and FLAT^n^ (direct biomass MS/MS for fragmentation and structural analysis of lipid A in negative ion mode). For FLAT procedures, each bacterial strain was picked from the sample vials and the bacterial slurry was directly smeared on the ITO-glass slide. For each FLAT, only 1 µl of slurry was used. To reduce phospholipid signals from the mass spectra, ethanol prewashing was conducted for 30 sec. Briefly, the sample-loaded ITO-glass slide was dipped into ethanol and then removed for ethanol evaporation. Next, FLAT was conducted according to the previous literature (Yang et al., 2023). Briefly, 1 μl of the prepared citrate buffer solution (0.2 M citric acid, 0.1 M trisodium citrate, pH 3.5) was deposited onto the sample spot on the ITO slide. The plate was incubated in a humidified, closed glass chamber for 30 min at 110°C. After heating, the ITO slide was removed from the chamber, cooled, and the plate was thoroughly washed with water using a pipettor several times and left to dry on the laboratory bench. For FLAT^n^ experiments, the precursor ion (lipid A) was chosen by targeted *m/z* value to the hundredth of a mass unit. Typical isolation width and collision energy were set to 4–6 *m/z* and 100–110 eV, respectively, and *m/z* scan range of 200 to 3000. Agilent ESI Tune Mix was used to perform calibration of the *m/z* scale. MALDI parameters in qTOF were optimized to maximize intensity by tuning ion optics, laser intensity, and laser focus. All spectra were collected at 104 µm laser diameter with beam scan on, using 800 laser shots per spot and 70 and 80% laser power. Both MS and MS/MS data were collected in negative ion mode using 10 mg/ml of norharmane (NRM) matrix in 1:2 MeOH: CHCl_3_ (v:v) for ionization of lipid A. All MALDI (timsTOF) MS and MS/MS data were visualized using mMass (Ver 5.5.0, www.mmass.org). Picking the peaks was conducted in mMass by following parameters: S/N threshold: 3.0, Absolute Intensity threshold: 1.0, Relative Intensity threshold: 1.0%, Picking height: 50, Apply baseline, and Apply smoothing. Identification of all fragment ions were determined based on exact mass as predicted by ChemDraw Ultra (Ver10.0, PerkinElmer, Waltham MA).

### 2.5 MALDI-TOF analysis of lipid A

The lipid A from planktonic cells was released from LPS samples by the method of Caroff (Caroff et al., 1988). Briefly, LPS samples were dissolved in 1% SDS in 10 mM sodium acetate buffer (pH 4.5) and incubated at 100˚C for 75 min with constant stirring. The samples were immediately frozen and lyophilized overnight. The reaction mixture was washed three times with acidified EtOH multiple times to remove any traces of SDS and the resulting lipid A was resuspended in water and purified by chloroform/water extraction (1:1; v/v). After the final wash, the chloroform layer was removed, evaporated, dissolved in 20 µl of chloroform-methanol solution (3:1; v/v) and then mixed with 0.5 M 2,4,6-trihydroxyacetophenone matrix (Sigma) in methanol (1:1 ratio; v/v) and transferred onto a MALDI plate. The spectra were acquired in negative and positive reflector ionization modes ([M-H]^−^ and [M+Na]^+^, respectively) using an Applied Biosystems AB 4800 proteomics analyzer. The spectra were calibrated in both ionization modes using Angiotensin I as the external standard. For biofilm cells, the experiment was performed as described above, except that the incubation at 100˚C with SDS was performed only for 55 min, and the spectra were acquired using a Bruker MALDI-ToF MS rapifleX^®^ Tissuetyper. The spectra were calibrated in both ionization modes using Angiotensin I as the external standard.

### 2.6 Glycosyl and fatty acid composition analysis

The glycosyl and the fatty acid analysis was performed by combined gas chromatography-mass spectrometry (GC-MS) of the O-trimethylsilyl (TMS) methyl derivatives produced from the sample by acidic methanolysis with 1 M HCl-methanol at 80°C for 18 h, in the presence of an internal standard of *myo*-inositol (York et al., 1986). The TMS method also identified methyl esters of the straight-chain and hydroxylated fatty acids constituting LPS [fatty acid methyl esters (FAME and TMS-FAME, respectively)]. The TMS and FAME derivatives were analyzed on a Hewlett-Packard HP5890 gas chromatograph equipped with mass selective detector 5970 MSD using an EC-1 fused silica capillary column (30 m, 0.25-mm inner diameter). The oven temperature was set at 80°C for 2 min, then increased to 140°C at 20°C/min with 2 min hold, and to 200°C at 2°C/min, followed by an increase to 250°C at 30°C/min with 5 min hold.

### 2.7 Lipid extraction and liquid chromatography-mass spectrometry analysis

Bacterial cultures were cultivated as described above. Cell pellets were harvested and freeze dried. The dried pellets (100 mg) were dissolved in a small volume of methanol and vortexed. The samples were centrifuged and transferred to autosampler tubes. Analysis of lipids was performed utilizing mass spectrometric instrumentation located in the Center for Environmental Sciences and Engineering at the University of Connecticut, Storrs, as previously described (Guido et al., 2024). An ACE Excel 2 C18 column (75 x 2.1 mm), maintained at 50°C, was employed for analyte separation, with a sample injection volume of 10 µl on a 20 µl loop. The mobile phase comprised two solvents: solvent A (10 mM ammonium formate, 0.1% formic acid in 40% water/60% acetonitrile) and solvent B (10 mM ammonium formate, 0.1% formic acid in 90% isopropyl alcohol/10% acetonitrile), utilized for gradient elution. Initially, a flow rate of 75% solvent A was maintained for 3 min, followed by a linear increase to 100% solvent B over the next 5 min. The column was then reconditioned to the initial state for 1 min. The total run time was 10 min with a constant flow rate of 0.4 ml/min. The detection and quantification of lipid analytes were performed in negative ESI-MS/MS mode (MRM) using the Sciex instrument. Parameters for the Sciex500 QTOF TurbolonSpray included a curtain gas of 30, ion source gas 1 at 55, ion source II at 60, and a temperature of 500°C. The declustering potential was set at -80V, and collision energy ranged from -8 to -60V. Statistical analysis for quantification was carried out using Sciex500 software. Lipid classes were identified based on characteristic retention times of purified or synthetic standards, quantifying either the characteristic molecular parent ion or dominant MS/MS ion transitions (MRM). The experiment was repeated three times for statistical analysis.

### 2.8 SDS and native polyacrylamide gel electrophoresis and LPS staining

SDS-PAGE analysis of purified LPS (2 µg/well) was performed using 16% Novex™ Tris-Glycine Mini Protein Gels (Invitrogen) at a constant current of 100 V for 150 min. Native PAGE was performed using NativePAGE™ 4 to 16% Bis-Tris gels (Cat No. BN1002BOX) at a constant current of 150 V for 30 min. For polymyxin B-treated LPS samples, purified LPS samples were incubated with 20x the amount of polymyxin B (InvivoGen) at 37°C overnight. Gels were stained employing the Pro-Q™ Emerald 300 Lipopolysaccharide Gel Stain Kit (Invitrogen), following the manufacturer’s protocol. Briefly, the gel underwent fixation at RT by immersion in 100 ml of fixing solution (50% methanol and 5% acetic acid in water) with gentle agitation for 45 min, twice. Subsequently, it was washed with 100 ml of wash solution (3% glacial acetic acid in water) for 15 min, twice. The gel was then incubated in 25 ml of oxidizing solution containing periodic acid in 3% acetic acid with gentle agitation for 30 min. The washing step was repeated twice. A fresh Pro-Q^®^ Emerald 300 Staining Solution was prepared by a 50-fold dilution of the Pro-Q^®^ Emerald 300 stock solution. This dilution involved adding 6 ml of DMF to the vial containing the Pro-Q^®^ Emerald 300 reagent of the kit and gently mixing it. The gel was then incubated in the dark in 25 ml of Pro-Q^®^ Emerald 300 Staining Solution with gentle agitation for 90 min. The Pro-Q^®^ Emerald 300 stain excites at about 280 nm and emits at roughly 530 nm. For visualization, the stained gel was observed using a gel doc (BIO-RAD) SYBR Safe program, which provides optimal conditions for viewing.

The Pierce™ Silver Stain kit for Mass Spectrometry (Thermo Scientific) was utilized following the manufacturer’s instructions. Following the fixing step, described above, a 30-min treatment with periodic acid in 3% acetic acid at RT was conducted to oxidize the bands. Briefly, the procedure involves the following sequential steps: washing the gel in ultrapure water for 5 min, twice; immersing the gel in Fixing Solution (6:3:1 water: ethanol: acetic acid) for 40 min, repeated twice at RT; and subsequent washing of the gel with 10% ethanol for 5 min, twice. The gel was then treated with 20 ml of oxidation solution from the Pro-Q™ Emerald 300 Lipopolysaccharide Gel Stain Kit, with gentle agitation for 30 min. This was followed by washing the gel with 10% ethanol for 10 min, twice, and a further wash in ultrapure water for 5 min, twice. The subsequent steps involved incubating the gel in a Sensitizer working solution (a mix of 50 µl Sensitizer with 25 ml water) for precisely 1 min, followed by two changes of ultrapure water for 1 min each. Thereafter, a mixture of 1 part Silver Stain Enhancer with 100 parts Silver Stain was added to the gel. The gel was incubated for 5 min. To prepare the developer working solution, 1 part Silver Stain Enhancer was mixed with 100 parts Silver Stain Developer. The gel was swiftly washed twice with ultrapure water for 20 sec each and then treated with the developer working solution. The gel was incubated until the desired band intensity appeared (~2–3 minutes). Upon achieving the desired intensity, the developer working solution was replaced with Stop Solution (5% acetic acid). The gel was briefly washed with distilled water and the image was analyzed using an iBright1500 Imaging System (ThermoFisher Scientific).

### 2.9 Western blot

For Western immunoblot analysis, the separated LPS bands on gels were transferred to PVDF membranes (polyvinylidene fluoride; Invitrogen) using the iBlot 2 dry system (ThermoFisher Scientific) in a three-step process: 15V for 2 min, 20V for 5 min, 23V for 30 sec. Subsequently, the membranes were washed 3 times, each for 5 min, in a pH 8.0 buffer (TTBS) comprising 20 mM Tris, 500 mM NaCl, and 0.1% Tween-20. Membranes were blocked with 5% non-fat milk in TTBS overnight at 4˚C, followed by three additional washes with TTBS buffer, each for 5 min. The membranes were then incubated at RT for 3.5 h with primary antibodies in 1% non-fat milk in TTBS: either anti-LPS mAB IB5 (from Dr. J. Potempa, University of Louisville, KY, USA) at a dilution of 1:1000 or a mouse monoclonal anti-*Pg* LPS antibody (Cat No. SAB4200834; Sigma Aldrich) at a dilution of 0.3:1000. Following incubation with the antibodies, the membranes were washed and treated with suitable secondary antibodies in the same buffer for 1 h and 15 min at RT: either mouse IgG horseradish peroxidase-conjugated antibody (Cat No. HAF007; Bio-Techne, USA) at a dilution of 1:1000 or goat anti-mouse IgG2b cross-adsorbed secondary antibody, HRP (Cat No. M32407; Invitrogen) at a dilution of 0.4:1000 respectively. Subsequently, the membranes underwent six 10-min washes with TTBS. Then, a working solution of SuperSignal™ West Pico PLUS chemiluminescent substrate (Cat No. 34580; Thermo Scientific) was prepared and applied to the membrane for imaging using iBright1500 Imaging System (ThermoFisher Scientific).

### 2.10 ELISA quantification of LPS

Purified LPS samples were assessed by Enzyme-Linked Immunosorbent Assay (ELISA). Briefly, a minimum of 50 ng LPS was added to each well, in triplicate, in 50 mM Carbonate/Bicarbonate buffer, pH 9.6. and was left to incubate overnight at 4°C. The wells were washed three times in PBS buffer containing 0.05% Tween-20 at RT. Following this, a blocking solution containing 5% non-fat milk in PBS (Cat No. M17200; RPI Research Products International) was applied to each well and left at RT for 1 h, after which it was washed thrice with the same washing buffer. The primary antibody, mouse monoclonal anti-*Pg* LPS antibody, (Cat No. SAB4200834; Sigma Aldrich), was applied at a concentration of 1:5000 in PTB (phosphate-buffered saline with Tween-20) at RT and incubated for 1 h at 37˚C. Post-incubation, the wells were washed again with the washing buffer. Fifty μl of an appropriate secondary antibody conjugate (HRP linked) mentioned previously was added per well at a concentration of 1:2000 in PTB at RT. The samples were incubated for 1 h at 37˚C and washed four times with the washing buffer. The reaction was visualized using a TMB substrate (Cat No. 34022; Thermo Scientific) at RT. The reaction was terminated by adding 50 μl of 1 N HCl, and the color measured at 450 or 630 nm wavelength in a SpectraMax iD3 (Molecular Devices).

### 2.11 Endotoxin activity assays

Endotoxin activities of LPS samples were determined using a Pierce^™^ Chromogenic Endotoxin Quant Kit (Thermo Scientific) which uses the horseshoe crab clotting mechanism to determine endotoxin content. Serially diluted LPS samples, with concentrations ranging from 10 ng/ml to 100 pg/ml, were assayed for endotoxin levels by comparison with *E. coli* (0111:B4) LPS standards (0.1–1.0 U/ml) in a 96-well microplate (**Supplementary Figure 11**). A straight-line equation generated from the *E. coli* LPS standards was used to calculate endotoxin activity in each LPS sample. To determine the effect of *Pg* LPS samples on *E. coli* endotoxin activity, 0.5 U/ml of *E. coli* LPS (from the kit) was preincubated with an equal volume of *P. gingivalis* LPS sample (1 ng/ml) or water (blank) for 10 min at 37°C. Equal aliquots of the preincubated samples were then used to determine endotoxin activities as outlined above. The experiments were performed at least thrice using duplicate wells.

### 2.12 Assessing the immunostimulatory potential of LPS samples

Immunostimulatory potential of the LPS samples was determined using HEK-Blue™ hTLR4 cells (InvivoGen) which uses secreted embryonic alkaline phosphatase (SEAP) as a reporter for TLR4 stimulation. Cells were plated in a T75 flask containing Medium A: DMEM (4.5 g/L glucose, 2 mM Glutamine), 10% FBS, 1X Antibiotic-Antimycotic (Gibco™), and 50 µg/ml Normocin (InvivoGen). At 70–80% confluency, cells were rinsed with PBS, detached using TrypLE™ Express Enzyme (Gibco), and neutralized with Medium A. Cells were centrifuged and gently resuspended in 5 ml of HEK-Blue Detection medium (InvivoGen). After assessing for cell detachment, cells were counted using a Cellometer Auto T4 (Nexcelom Bioscience, Lawrence, MA, USA) and then diluted to 1.5 x 10^6^ cells/ml in Detection medium. A total of 180 ul of the cell suspension was dispensed into each well of a microtiter plate containing various concentrations of the LPS samples – 0.05 µg/ml, 0.5 µg/ml, 1 µg/ml, and 5 µg/ml. As a positive control, *E. coli* LPS (Cat# LPS-EB; InvivoGen) was used. Wells containing cells alone without LPS samples were used as negative controls. To assess if the observed effects were TLR4-specific, LPS samples were similarly tested in HEK-293 Null Blue (InvivoGen) cells.

### 2.13 Statistical analysis

The experiments were conducted with a minimum of three biological replicates. GraphPad Prism version 9.2.0 was employed for statistical analyses. Pairwise comparisons were assessed using Student’s *t*-test. Statistical significance was determined at a probability value of < 5.0% (*P-*value < 0.05). For other data, the Shapiro-Wilk test (*P* > 0.05) was used to evaluate the normality of the distribution of the data followed by one-way ANOVA. For ELISA assays, *P*-values were calculated using analysis of covariance (ANCOVA). Differences in the data were considered significant when the *P* value < 0.05.

## 3 Results

### 3.1 C-di-AMP synthesis links bioenergetic status and LPS-modulating factors

**Figure 1:**
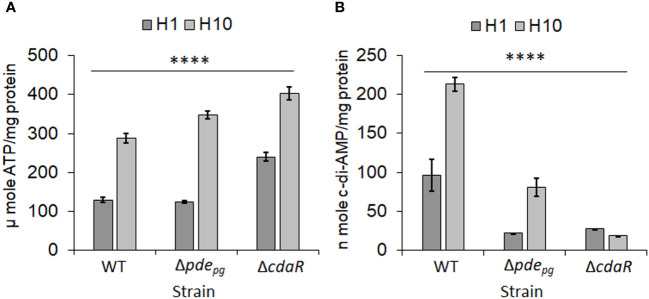
Analysis of low and high hemin conditions on cellular concentrations of ATP and c-di-AMP in *P. gingivalis* 381 (WT). **(A)** Cellular concentration of ATP significantly increases with higher hemin concentrations. The mutants Δ*pde_pg_* and Δ*cdaR* exhibit higher ATP accumulation compared to the WT. **(B)** The increase in hemin concentration significantly elevates the c-di-AMP pool in WT *P. gingivalis*, while the mutants, notably Δ*cdaR*, display defects in the incorporation of ATP into c-di-AMP molecules. Graphs represent the mean ± SE (three biological replicates) of ATP or c-di-AMP concentration which were analyzed using Shapiro-Wilk test (P > 0.05) followed by one-way ANOVA test (****P < 0.0001). Standard curves are presented in **Supplementary Figure S11**. WT, wild type; H, hemin (1 or 10 µg/ml).

Previously, we demonstrated that both *pde_pg_* and *cdaR* genes are required for the incorporation of ATP into c-di-AMP. This was evidenced by the fact that while pyruvate supplementation significantly increases cellular ATP levels in the Δ*pde_pg_* and Δ*cdaR* mutants compared to WT, it significantly reduces cellular c-di-AMP levels in the mutants (Moradali et al., 2022). Given that the mutants Δ*pde_pg_* and Δ*cdaR* exhibit defects in regulating cellular c-di-AMP and ATP and display significant alterations in the LPS-driven phenotype (Moradali et al., 2022), we hypothesized that this signaling system couples the bioenergetic status of the cells and the regulation of LPS profile upon alterations in the availability of bioenergetic substrates. To test this hypothesis, we used low (1 µg/mL) and high (10 µg/mL) concentrations of hemin, a key bioenergetic factor for *P. gingivalis* growth known to induce alterations in the LPS profile (Simpson et al., 2000; Al-Qutub et al., 2006; Coats et al., 2009) and assessed the impact of the two hemin conditions on cellular ATP and c-di-AMP levels of the WT and the two mutant strains. Our data revealed that the increase in hemin availability significantly enhances ATP production, subsequently elevating the cellular concentration of c-di-AMP in the WT. However, the mutants accumulate ATP significantly more than the WT (**Figure 1A**), consistent with our previous findings demonstrating that c-di-AMP signaling is required for regulating the bioenergetic status of the cells in response to the utilization of nutrients, such as exogenous pyruvate (Moradali and Davey, 2021; Moradali et al., 2022). In contrast, elevated hemin levels significantly increased c-di-AMP concentrations in the WT strain and to a lesser extent in Δ*pde_pg_*; however, it significantly decreased c-di-AMP concentration in the Δ*cdaR* mutant (**Figure 1B**). Consistent with our previous data (Moradali et al., 2022), this observation in mutants highlights the crucial roles played by PDE_pg_ and CdaR in facilitating the incorporation of ATP into c-di-AMP molecules, particularly in response to dynamic shifts in bioenergetic changing conditions, such as hemin and pyruvate availability. Of note, our previous study ruled out the polar effects of the deletion of *pde_pg_* or *cdaR* on nearby genes, as complementation with the relevant genes restored c-di-AMP levels to WT concentrations (Moradali et al., 2022). However, this was not utilized in the present study as an antibiotic resistance cassette is required for the complementation of the mutants and this will significantly impact the antibiotic selective pressure on *P. gingivalis* physiology and LPS structure. Given that alterations in the cellular c-di-AMP pool can change the integrity of *P. gingivalis* cell envelope, notably affecting the protective function and immunoreactivity of LPS (Moradali et al., 2022), we next sought to determine whether dysregulation of c-di-AMP signaling compromises *P. gingivalis* ability to modulate the LPS profile in response to changes in the bacterium’s bioenergetic states.

### 3.2 Dysregulation of c-di-AMP signaling significantly compromises the regulation of the lipid A profile

We investigated the role of c-di-AMP signaling in the regulation of the LPS profile by assessing the capabilities of *P. gingivalis* WT and mutant strains in regulating ‘relative abundances’ of major lipid A variants, as well as relevant core oligosaccharide region, and total LPS polysaccharide. Given *P. gingivalis* capability to produce different lipid classes (Nichols et al., 2004, 2006; Moye et al., 2016; Kleiboeker et al., 2023), we sought to confirm the specific regulatory role of c-di-AMP signaling on the LPS profile and regulation of other lipid classes. Hence, we analyzed distinct lipid classes in the WT, as well as the Δ*pde_pg_* and Δ*cdaR* mutants, cultivated at low and high hemin concentrations. Quantification and statistical analysis of various lipid classes (Nichols et al., 2011; Clark et al., 2013; Nichols et al., 2020), including dihydroceramides, phospholipids, and serine-glycine lipopeptides, did not reveal a significant difference in their relative abundance between WT and mutants (**Supplementary Figure 1**; **Supplementary Table 1**). These data further confirm that other lipid classes were not affected in the mutants which display shifts in c-di-AMP signaling.

**Figure 2:**
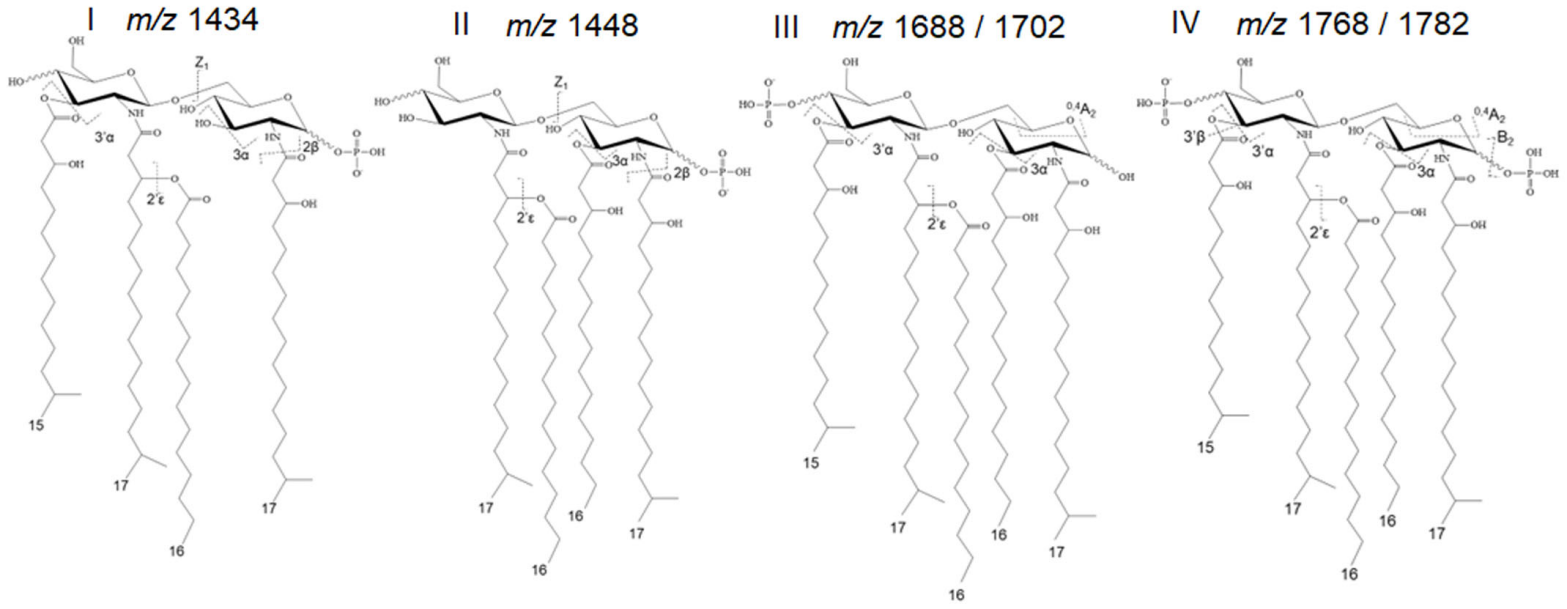
Predominant lipid A variants in *P. gingivalis* 381. The FLAT*^n^* technique has enabled the accurate calculation of the mass and relative abundance of structurally varied lipid A isoforms upon the analysis of purified LPS samples or colonies. Additional information on these calculations is provided in **Supplementary Figure S3**.

It is important to highlight that the precise chemical composition of the entire spectrum of *P. gingivalis* LPS variants remains unknown. Thus, our data interpretation has been limited to identifiable and interpretable mass spectrometry data. Further, the proper interpretation of LPS structural and functional data is influenced by different conditions, including bacterial growth conditions, chemical treatments during purification, the method of analysis, and various co-extracted lipidic contaminants. It is noteworthy to mention that strain 381 used in this study does not produce capsular polysaccharides, eliminating potential interference with the interpretation of data (Graham et al., 1991; Chen et al., 2004; Aduse-Opoku et al., 2006). Prior to studying the LPS profile of the select strains, we optimized our methodology by combining conventional MALDI-ToF MS techniques with a novel approach known as tandem MS version of the fast lipid analysis technique (FLAT*^n^*) (Yang et al., 2022). Leveraging the existing knowledge about *P. gingivalis* lipid A profile (Kumada et al., 1995; Ogawa et al., 2002, 2007), we theoretically calculated and proposed the predicted *m/z* for the most common *P. gingivalis* lipid A variants based on available MALDI-ToF MS data. Essential information for deciphering the structures is provided in **Supplementary Table 2**. The FLAT*^n^* technique has enabled the accurate calculation of the mass (Δ ± 0.02 Da) and relative abundance of structurally related lipid A isoforms, allowing for *in situ* lipid analysis on bacterial colonies or intact LPS samples purified from the cells. To test the reproducibility and effectiveness of the FLAT*^n^* technique in deciphering the complexity of *P. gingivalis* lipid A profile, we initially cultivated WT cells under conditions similar to those described in pioneering studies (Al-Qutub et al., 2006; Coats et al., 2009). We conducted analyses on both intact highly pure LPSs (FLAT*^n^*/LPS_pure_) and on the colonies (FLAT*^n^*/*in situ*). Notably, the FLAT*^n^*/LPS_pure_ analysis provided a more accurate insight into prominent *P. gingivalis* lipid A variants I-IV, appearing between *m/z* 1400 and 1800, demonstrating reproducibility when compared to previous studies (Al-Qutub et al., 2006; Coats et al., 2009). It also revealed a precise and distinct relative abundance of these prominent lipid A variants I-IV under high and low hemin conditions, as illustrated in **Figure 2**, **Supplementary Figures 2** and **3**. However, the FLAT*^n^*/*in situ* was not able to detect a full range of lipid A variants when compared to the FLAT*^n^*/LPS_pure_ approach (**Supplementary Figure 4**). Importantly, the FLAT*^n^*/LPS_pure_ eliminated potential negative impacts that could arise during the lipid A release process, potentially resulting in difficulties in observing a full range of lipid A variants in conventional approaches. Accordingly, the FLAT*^n^*/LPS_pure_ approach enabled us to detect and measure accurately the intensity of *bis*-phosphorylated pentaacyl variants (*m/z* 1768 and 1782) - the primary and progenitor form of lipid A in *P. gingivalis*, known for its immunostimulatory potency among other variants. These data underscore the robustness and precision of our approach in determining the role of c-di-AMP signaling in controlling the lipid A profile under distinct bioenergetic statuses of the cells. Additionally, we performed conventional lipid A release on purified LPS samples, followed by MALDI-ToF MS analysis (negative- and positive-ion modes). We proved that this conventional approach to release and purify the lipid A component of *P. gingivalis* LPS proved inefficient, likely due to specific chemical modifications causing a significant loss of major lipid A variants, particularly the pentaacyl-P2 (IV; *m/z* 1768 and 1782) variants (**Supplementary Tables 3**, **4**). Consequently, this approach failed to provide an accurate interpretation of the lipid A profile. Overall, these findings further highlight the efficiency of the FLAT*^n^*/LPS_pure_ approach in our study.

**Figure 3:**
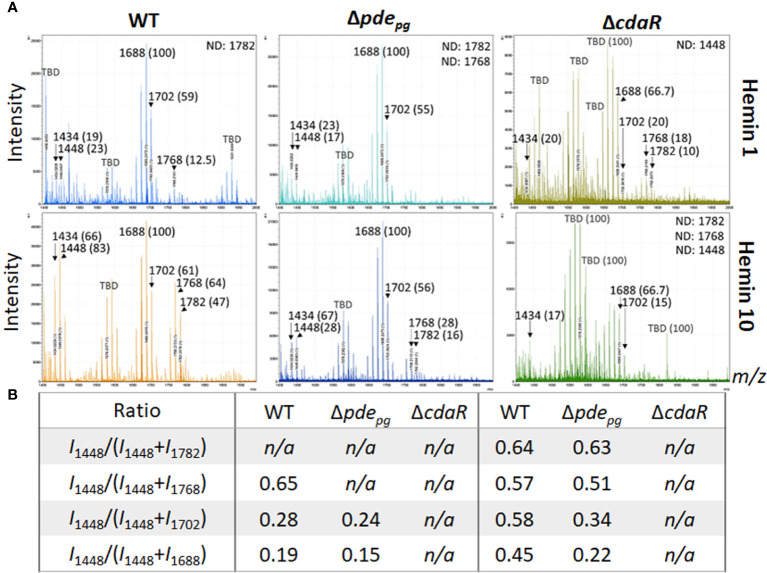
Comparison of relative abundances of the predominant lipid A variants in the LPS profiles of the WT, Δ*pde_pg_* and Δ*cdaR* mutants upon hemin availability. Data show that c-di-AMP dysregulation in mutants compromises *P. gingivalis*`s ability to regulate the relative abundance of different lipid A variants in response to hemin concentrations. **(A)** The FLAT*^n^*/LPS_pure_ analysis of the lipid A profile of the WT and mutant strains under low and high hemin conditions. We calculated the *m/z* values (Δ ± 0.02 Da) of the six lipid A variants in the LPS profile of the WT. These include tetraacyl, P1 (I, 1434.02); tetraacyl, P1 (II, 1448.03); pentaacyl, P1 (III_a_, 1688.25); pentaacyl, P1 (III_b_, 1702.26); pentaacyl, P2 (IV_a_, 1768.21); pentaacyl, P2 (IV_b_, 1782.22). The identified lipid A variant with the highest intensity was designated as 100% to calculate the relative intensity of others (shown in parentheses), except when 100% intensity corresponded to a dominant *m/z* that was not identifiable (or TBD). **(B)** The ratios of major phosphorylated penta- and tetra-acylated variants. Notably, the Δ*cdaR* mutant exhibits a much lower abundance of the predominant pentaacyl-P1 (III) variants compared to the WT. WT, Wild type; TBD: to-be-determined; ND, Not detectable.

We applied our optimized approach, as described above, to gain deeper insights into the proposed c-di-AMP regulation of the lipid A profile. First, we performed the FLAT*^n^*/LPS_pure_ analysis on intact LPS samples purified from WT and mutant strains cultivated under low and high hemin conditions. This analysis revealed that the relative abundance of variants III (pentaacyl-P1; *m/z* 1688 and 1702) remained similar in the WT LPS profile under both low and high hemin conditions (**Figures 3A, B**). However, the relative abundances of variants I, II, and IV significantly changed, albeit proportionally, in response to different hemin conditions. In particular, the LPS of WT displayed a much greater percentage of the pentaacyl-P2 (IV) and tetraacyl-P1 (I, II) lipid A variants in response to high hemin availability. While the pentaacyl-P1 lipid A (III) variants seemed predominant in WT, alterations in their relative abundance were independent of variants I, II, and IV (**Figures 3A, B**). When considering the mutants from high hemin conditions, the lipid A profile of the Δ*pde_pg_* mutant resembled that of the WT with respect to the pentaacyl-P1(III) variants; however, the relative abundances of the pentaacyl-P2 (IV) and tetraacyl-P1 (I, II) variants were lower in the Δ*pde_pg_* mutant compared to the WT. The changes in the lipid A profile of the Δ*cdaR* mutant were highly significant, with the relative abundances of all lipid A variants, notably the predominant pentaacyl-P1 (III) variants, being much lower compared to the WT under both hemin conditions. Additionally, the tetraacyl-P1 (II) and the pentaacyl-P2 (IV) variants were not detectable in this mutant under high hemin conditions (**Figures 3A, B**). This further supports the co-occurrence of variants I, II, and IV, while being independent of variant III.

**Figure 4:**
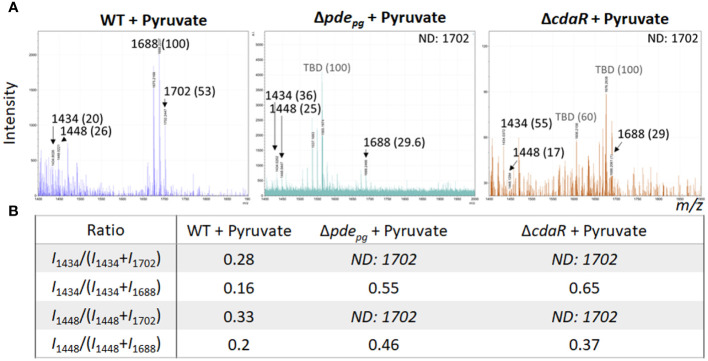
Comparison of relative abundances of the predominant lipid A variants in the LPS profiles of the WT, Δ*pde_pg_* and Δ*cdaR*mutants upon pyruvate availability. This data indicates that deletion of *pde_pg_* or *cdaR* gene compromises *P. gingivalis*` s ability to regulate the relative abundance of major lipid A variants in response to pyruvate availability. **(A)** The FLAT*^n^*/LPS_pure_ analysis of the lipid A profile of the WT, Δ*pde_pg_* and Δ*cdaR* mutant upon pyruvate supplementation. We calculated the *m/z* values (Δ ± 0.02 Da) of the six lipid A variants in the LPS profile of the WT. These include tetraacyl, P1 (I, 1434.02); tetraacyl, P1 (II, 1448.03); pentaacyl, P1 (III_a_, 1688.25); pentaacyl, P1 (III_b_, 1702.26); pentaacyl, P2 (IV_a_, 1768.21); pentaacyl, P2 (IV_b_, 1782.22).The identified lipid A variant with the highest intensity was designated as 100% to calculate the relative intensity of others (shown in parentheses), except when 100% intensity corresponded to a dominant *m/z* that was not identifiable (or TBD). **(B)** The ratios of major assignable lipid A variants. WT, Wild type; TBD, to-be-determined; ND, Not detectable.

Given that PDE and CdaR are both required for the incorporation of ATP into c-di-AMP upon utilization of hemin (**Figure 1**) or exogenous pyruvate (Moradali et al., 2022), we hypothesized that this signaling pathway plays a crucial role in coupling the bioenergetic state of the cells and the regulation of the specific lipid A variants. To test this hypothesis, we assessed the impact of exogenous pyruvate, as a source of carbon and energy (Moradali and Davey, 2021; Moradali et al., 2022), on the lipid A profile of WT, Δ*pde_pg_* and Δ*cdaR* mutants. Since the Δ*cdaR* mutant shows a growth defect in the pyruvate-supplemented medium, as observed in our previous study (Moradali et al., 2022), conducting the FLAT*^n^*/LPS_pure_ analysis was not feasible. Therefore, we employed FLAT*^n^*/*in situ* analysis to test our hypothesis, with an emphasis on the detectable variants, notably the predominant pentaacyl-P1 variants. Our findings demonstrated that pyruvate supplementation significantly increases the relative abundance of the predominant pentaacyl-P1 variants in the WT, while this effect was abolished in both mutants compared to the WT (**Figures 4A, B**). Moreover, lipidomic analysis showed that utilization of exogenous pyruvate did not change the relative abundance of other lipid classes in the WT strain (**Supplementary Figure 5**). These data further support the crucial role of c-di-AMP signaling in linking the bioenergetic status and the LPS profile in *P. gingivalis*.

The FLAT*^n^*/LPS_pure_ data further revealed significant signals greater than m/z 1871, showing dissimilar intensity and relative abundance between WT and mutants (**Supplementary Figure 6**). In theory, signals in this region of *P. gingivalis* LPS could be attributed to the lipid A/core oligosaccharide component and its chemical modifications. As suggested by *P. gingivalis* genomic data available in the BioCyc database (https://biocyc.org), these modification forms include ethanolamine-modified lipid A, phosphoglycerol-lipid A, allosaminyl-phosphoglycerol-lipid A, and α-D-mannosyl-[allosaminyl]-phosphoglycerol-lipid A, representing the intermediate forms of the lipid A/core oligosaccharide. These structural modifications potentially result in the inclusion of additional 3–5 α-D-mannopyranosyl residues into the Kdo/core oligosaccharide components, thereby significantly altering LPS function during infection (Kubo et al., 2004; Rangarajan et al., 2008).

### 3.3 Dysregulation of c-di-AMP signaling alters fatty acid and glycosyl compositions in the LPS profile of *P. gingivalis*

**Figure 5:**
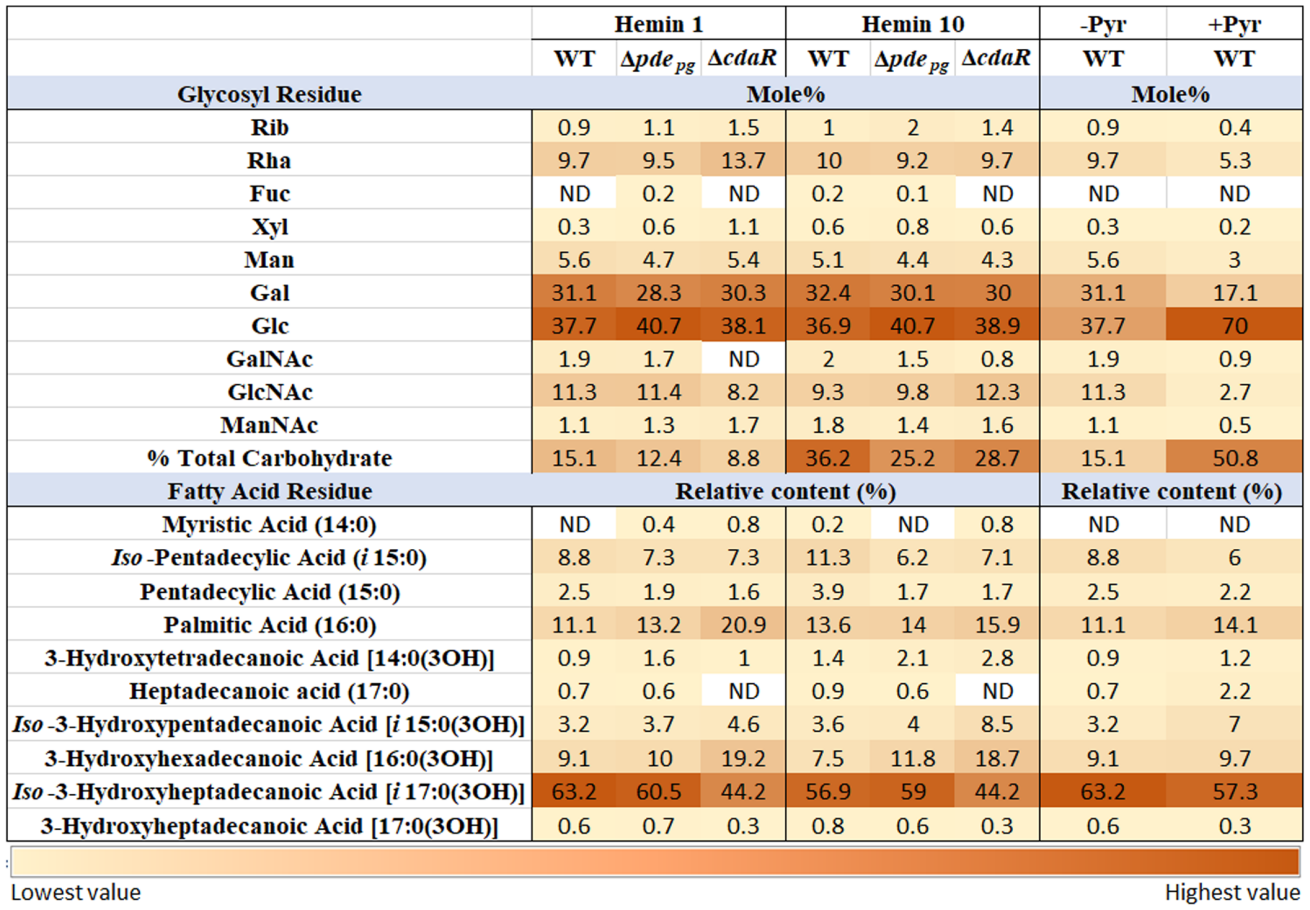
Relative abundance of glycosyl and fatty acid residues in the LPS profile of WT and Δ*pde_pg_* and Δ*cdaR* mutants upon changing bioenergetic status. Notably, GC-MS data revealed the complete absence or a considerable reduction in GalNAc residues within the LPS of Δ*cdaR*, particularly evident under low hemin condition. Our data revealed the predominance of longer fatty acid chains in *P. gingivalis* LPS. Comparative analysis of the fatty acid profile of the WT and mutants revealed two-fold increase in 3-hydroxyhexadecanoic acid [16:0(3OH)], absence of heptadecanoic acid (17:0), and a significant reduction of *Iso*-3-hydroxyheptadecanoic acid [*i*17:0(3OH)] and 3-hydroxyheptadecanoic acid [17:0(3OH)] in the Δ*cdaR* mutant. The augmented bioenergetic state of the cells upon pyruvate utilization results in a notable increase in the glucose content of the polysaccharide, accounting for 70% of the total sugars, while galactose is reduced by almost half. Additionally, it reduces 3-hydroxyheptadecanoic acid [17:0(3OH)] and *Iso*-3-hydroxyheptadecanoic acid [*i*17:0(3OH)], while increasing the abundances of heptadecanoic acid (17:0) and *Iso*-3-hydroxypentadecanoic acid [*i*15:0(3OH)]. Rib, Ribose; Rha, Rhamnose; Fuc, Fucose; Xyl, Xylose; Man, Mannose; Gal, Galactose; Glc, Glucose; GalNAc, N-acetylgalactosamine; GlcNAc, N-acetylglucosamine; ManNAc, N-acetylmannosamine; Pyr, Pyruvate.

In consideration of the distinct interactions of various constitutive elements of LPS macromolecules with host biological targets and receptors and given that prior investigations have primarily focused on variations within the lipid A profile, our study expanded the scope of analysis to investigate the impact of c-di-AMP signaling on the entire structure of *P. gingivalis* LPS. To achieve this, we conducted analyses of sugar and fatty acid constituents on the isolated LPS samples of WT and mutant strains. Our data revealed the predominance of longer fatty acid chains in *P. gingivalis* LPS (**Figure 5**; **Supplementary Figure 7**). Notably, *Iso*-3-hydroxyheptadecanoic acid [*i*17:0(3OH)] constitutes ~60% of the total fatty acid profile. Additionally, palmitic acid [16:0] accounted for ~12%, *Iso*-pentadecyclic acid (*i*15:0) ~9%, 3-hydroxyhexadecanoic acid [16:0(3OH)] ~9%, and *Iso*-3-hydroxypentadecanoic acid [*i*15:0(3OH)] ~3%. Comparative analysis of the fatty acid profile of the WT and the Δ*cdaR* mutant revealed two-fold increase in 3-hydroxyhexadecanoic acid [16:0(3OH)], absence of heptadecanoic acid (17:0), and a significant reduction of *Iso*-3-hydroxyheptadecanoic acid [*i*17:0(3OH)] and 3-hydroxyheptadecanoic acid [17:0(3OH)] in the mutant (**Figure 5**; **Supplementary Figure 7**).

Furthermore, our data revealed that in the WT LPS, different isoforms of glucose and galactose collectively account for 60%-70% of the total glycosyl residues in the polysaccharide components of LPS, while rhamnose constitutes approximately 10% of that. However, the mutants did not display a significant shift in the predominant sugar constituents compared to WT LPS. Of notable significance is the complete absence or a considerable reduction in N-acetylgalactosamine (GalNAc) residues within the LPS of Δ*cdaR*, particularly evident under low hemin condition (**Figure 5**; **Supplementary Figure 7**). This occurrence may potentially result in the loss of formation or elongation of the O-antigen polysaccharide and other glycolipid chains in bacteria (Paramonov et al., 2009; Whitfield et al., 2020). Furthermore, a significant distinction was found to exist between the LPS polysaccharide of the mutants and that of the WT, as two molecular forms of the functionally and pathologically significant ManNAc residues are discernible in Δ*pde_pg_*, while in Δ*cdaR*, a dissimilar form of ManNAc can be observed in comparison to the WT (**Supplementary Figure 7**). Of note, we could not detect the 3-deoxy-D-manno-oct-2-ulosonic acid (Kdo) residue signal in WT and mutants (**Figure 5**; **Supplementary Figure 7**) which supports the existence of hitherto unknown chemical modifications in this region of *P. gingivalis* LPS that awaits further investigation.

Moreover, our findings revealed that the augmented bioenergetic state of *P. gingivalis* cells upon pyruvate utilization results in a notable reduction of 3-hydroxyheptadecanoic acid [17:0(3OH)] and *Iso*-3-hydroxyheptadecanoic acid [*i*17:0(3OH)] and increase of heptadecanoic acid (17:0) and *Iso*-3-hydroxypentadecanoic acid [*i*15:0(3OH)]. Additionally, pyruvate utilization significantly increases the glucose content of the polysaccharide, accounting for 70% of the total sugars, while galactose is reduced by almost half. In addition, N-acetyl-galactosamine, N-acetylglucosamine, and mannose content are reduced upon pyruvate supplementation. Consistent with these observations, pyruvate supplementation notably enhanced the intensity of high molecular weight bands (representing the entire spectrum of LPS macromolecules varying in polysaccharide length) in SDS-PAGE analysis, reflecting the observed increase in the percentage of total carbohydrates in LPS, without inducing a significant change in the relative percentage of other lipid classes in *P. gingivalis* (**Figure 5**; **Supplementary Figure 7B**). These data indicate that metabolic and bioenergetic status of this anaerobe primarily controls the structure and biological activities of LPS.

### 3.4 The c-di-AMP regulator CdaR is required for the production of unreported glycan chains associated with the LPS profile of *P. gingivalis*

**Figure 6:**
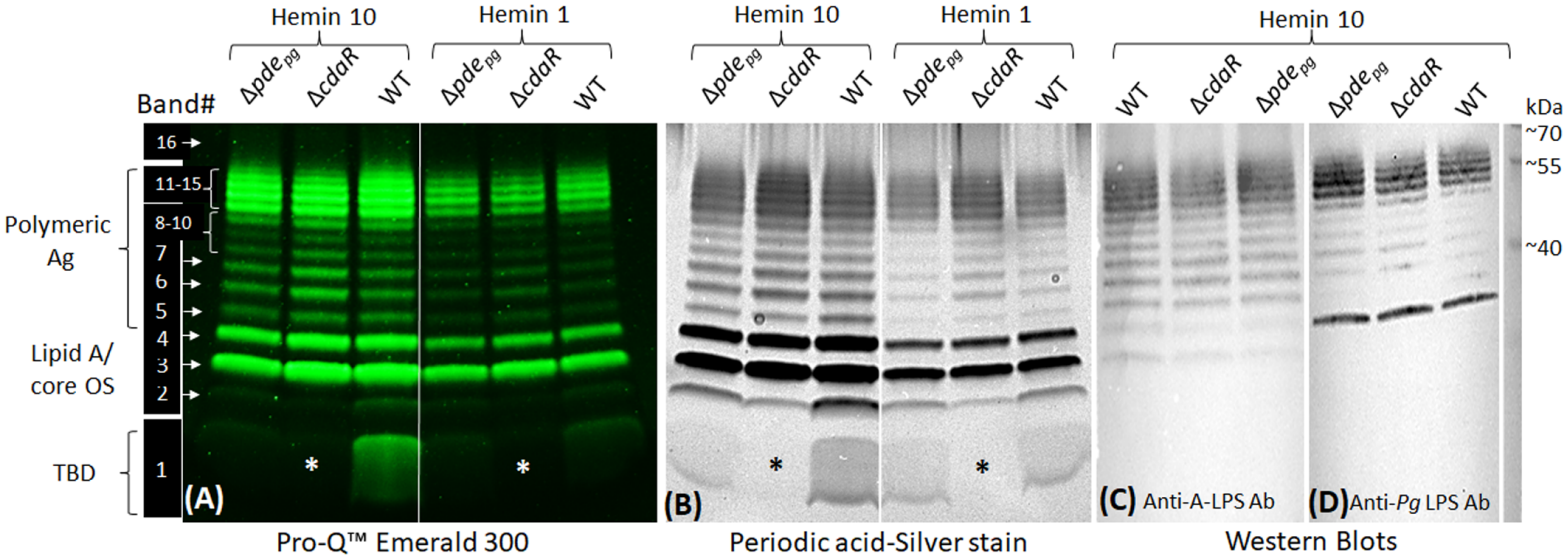
SDS-PAGE profile of LPS - polysaccharide staining and western blotting. **(A)** Use of the Pro-Q Emerald 300 LPS kit revealed 16 distinct bands in the LPS profile of WT and Δ*pde_pg_* mutant and 15 bands in Δ*cdaR* mutant. Band no. 1 was notably absent from the LPS profile of the Δ*cdaR* mutant, irrespective of hemin concentration (indicated by asterisks). **(B)** Band no. 1 was not detectable by standard silver staining techniques but can only be observed by silver staining subsequent to periodate oxidation treatment, suggesting glycan structures. **(C, D)** Western blot analyses of LPS samples probed with monoclonal antibodies 1B5 (also recognized as anti-A-LPS) and anti-*P. gingivalis* LPS antibody, respectively. 2 µg LPS was used in each lane. Ag, Antigen; OS, oligosaccharide; TBD, To be determined.

To confirm the impact of the shifts in c-di-AMP signaling on the biosynthesis of LPS variants, we performed polyacrylamide gel electrophoresis (PAGE) and western blot analyses. Typically, the profile of assembled LPSs contains various intermediates, exhibiting modal or ladder-like distribution of LPS bands in PAGE analysis. Using conventional silver-stained PAGE in the presence of sodium dodecyl sulfate (SDS) or sodium deoxycholate (DOC), we visualized 16 discernible bands, encompassing a spectrum of sizes from low to high molecular weight (MW) in the LPS profiles of the WT and mutant strains (**Figure 6**; **Supplementary Figure 8**). These particular bands were further confirmed as exhibiting immunoreactivity when probed with monoclonal antibodies (mAb), including 1B5 (specifically recognizing A-LPS) (Paramonov et al., 2005) and anti-*P. gingivalis* LPS antibodies (recognizing total LPS) (**Figure 6**). This observation indicated that the assembly process leading to the formation of LPS macromolecules of various sizes remained undisrupted in the mutant strains despite exhibiting significant shifts in the lipid A profile, as well as in fatty acid and glycosyl compositions. However, using Pro-Q Emerald 300 LPS gel stain kit, we unraveled an additional major low MW band, i.e., band no. 1, in the LPS profile of the WT and Δ*pde_pg_* strain which was notably absent from the LPS profile of the Δ*cdaR* mutant, irrespective of hemin levels/conditions. This band in the LPS profile was not detectable by standard silver staining techniques but can only be observed by silver staining after periodate oxidation treatment, suggesting glycosidic structures (**Figure 6**). Additionally, band no. 2, positioned directly above band no. 1, displayed similar intensity to band no. 1 across all sample variations, except in the Δ*cdaR* LPS in which it exhibited the lowest intensity. These particular bands did not exhibit immunoreactivity when probed with monoclonal antibodies (mAb), including 1B5 (Paramonov et al., 2005) and anti-*P. gingivalis* LPS antibodies (**Figure 6**). To provide some insights into the structure of this band, band no. 1 was extracted from the SDS-PAGE gel through passive elution using water and triethylamine (TEA), as described previously (Hardy et al., 1998; Pupo et al., 2000; Pupo and Hardy, 2007). Preliminary MS/MS data showed peaks at *m/z* 553, 841, 1129, 1195, 1417, and 1705 of the gel extracts (**Supplementary Figure 9**). These signals result from consecutive loss of the m/z 287 ion, indicating the polymeric nature of this component(s). Using the MassBank database (https://massbank.eu), the primary signal m/z 1195 in the TEA extract was predicted to be a glycan chain containing a tetrasaccharide sequence of hexoses. Additionally, GC-MS analysis of both TEA and water extractions revealed trace amounts of glycosyl components, including hexoses (*m/z* 73, 147, 173, 186, 191, 204, 217) and amino sugars (*m/z* 73, 96, 144, 173, 186, 204), as well as glycerol (*m/z* 73, 103, 117, 147, 205, 218) (Laine et al., 1988; Marschall et al., 1989; Cardelle-Cobas et al., 2009; Mookherjee et al., 2021; Veith et al., 2022). The *m/z* 168, 186, 204, 274, and 292 signals were attributed to the HexNAc and Neu5Ac ions (Toghi Eshghi et al., 2016). Also, glycerol and glucose signals matched the internal standards (**Supplementary Figure 9**). It is noteworthy that no common fatty acid residues of LPS or other lipid classes could be assigned to band no. 1. Although the exact identity and structure of band 1 remain to be elucidated, the distinctive attributes of this band, such as its low molecular weight, diffusive pattern, detection by the Pro-Q Emerald 300 LPS gel stain kit, and the presence of glucose, amino sugars, and glycerol content, suggest glycan chains of varying lengths associated with the LPS profile. Our present data reveal that the production of band 1 is tightly controlled by CdaR (**Figure 6**), and its association with the LPS profile remains to be clarified. Ongoing studies aim to fully characterize the structure of this component.

### 3.5 C-di-AMP signaling-driven LPS variants display varying biological activities and immunostimulatory potential

**Figure 7:**
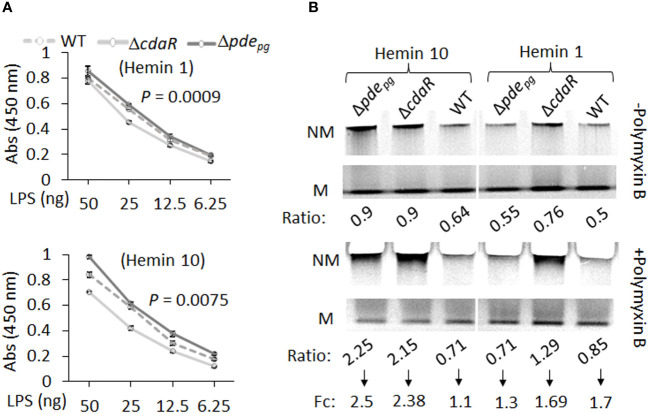
ELISA and gel mobility shift assays of LPS samples isolated from WT, Δ*pde_pg_* and Δ*cdaR* mutants cultivated under low and high hemin conditions. **(A)** ELISA assays were performed using anti-*P.gingivalis* LPS monoclonal antibody. The graph represents the mean ± SE of the immunoreactivity of each LPS sample (three biological replicates). The standard curve is presented in **Supplementary Figure S11**. *P*-values were calculated using analysis of covariance (ANCOVA) and indicate significant differences between the elevations. **(B)** Gel mobility shift assays were conducted under native electrophoresis conditions to investigate the relative abundance of charged and uncharged LPS variants in WT and mutants and their binding potential to the positively charged antibiotic polymyxin B (20 times the amount of LPS). The relative intensities of each band –’Non-mobile’ (NM; uncharged) or ‘Mobile’ (M; charged) were normalized to the high hemin (H10) WT band (**Supplementary Table S5**). Ratio is the intensity of each band (N or NM) as a percentage of total intensity (M+NM) and fold change (Fc) is NM ratio/M ratio. Each well contained 2 µg LPS.

We expected that the observed shifts in the composition of the LPS profile of the mutants could alter their biological activities, notably interaction with antimicrobials, endotoxin activity, and immunostimulatory potential. To complement our findings, we conducted an ELISA assay on the corresponding purified LPS samples. The results indicated that Δ*pde_pg_* LPS exhibited significantly higher immunoreactivity to anti-*P. gingivalis* LPS antibody compared to WT LPS, whereas Δ*cdaR* LPS exhibited lower reactivity (**Figure 7A**). Consequently, the outcomes derived from the analysis of purified LPS were consistent with previously reported data concerning the immunoreactivity of whole cell lysates of the mutants compared to WT (Moradali et al., 2022).

The LPS profile of *P. gingivalis* comprises a combination of uncharged and charged LPS molecules. The negative charge of *P. gingivalis* LPS variants, primarily dependent on the presence of phosphate groups and anionic residues of A-LPS, significantly influences intermolecular interactions and LPS biological activities. However, the relative abundance of these variants remained elusive in this species. To further assess the impact of dysregulated c-di-AMP signaling on this characteristic of the LPS profile, we conducted a gel mobility shift assay to assess the mobility of LPS samples, as well as the interaction between LPS and polymyxin B under “native” electrophoretic conditions. Polymyxin B, a positively charged antibiotic, is a widely used component in LPS binding studies (Knobloch et al., 2013; Manioglu et al., 2022). The comparative analysis of relative mobility shifts between polymyxin B-treated and untreated LPS samples run on a native gel is depicted in **Figure 7B** and **Supplementary Table 5**. In **Figure 7B**, the ‘Mobile’ (M) band represents negatively charged LPS variants that have been observed to migrate in the native gel, and the ‘Non-mobile’ (NM) band represents poorly charged LPS complexes that have not migrated sufficiently into the gel. Our data indicate that the relative abundance of charged variants (M) is nearly twice as high as uncharged variants (NM) in the WT LPS profile (**Figure 7B**). However, dysregulation of c-di-AMP signaling led to shifts in these ratios, resulting in mutants displaying an almost equal number of charged and uncharged LPS variants, particularly under high hemin conditions (**Figure 7B**). It is anticipated that the interaction of polymyxin B with band M will decrease its intensity with a concomitant increase in the intensity of band NM. The application of polymyxin B to WT LPS samples resulted in a 1.1-fold increase in the ratio of NM to M bands under high hemin conditions and a 1.7-fold increase under low hemin conditions. Notably, the LPS of the mutants, under high hemin conditions, exhibited a substantial fold increase, reaching up to 2.5-fold, in relative mobility shift. This observation in mutants and WT LPS suggests a critical role for c-di-AMP signaling in the regulation of the LPS profile, consequently contributing to effective evasion from biological binding agents, such as polymixin B.

**Figure 8:**
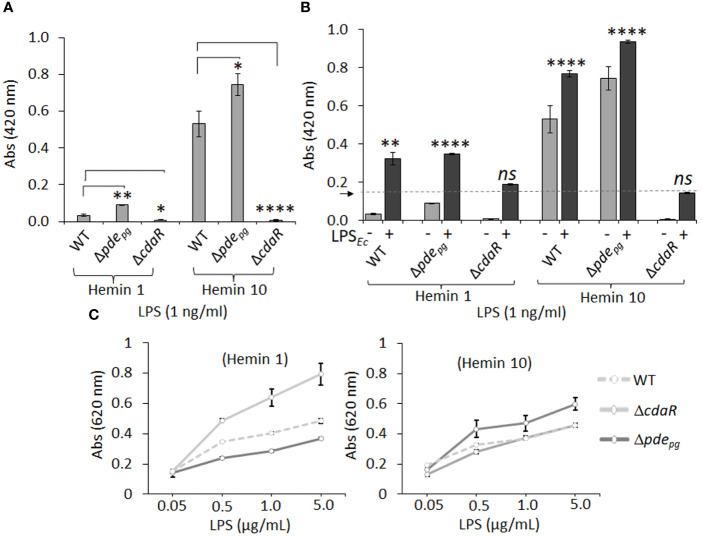
The endotoxin activity and immunostimulatory potential of the LPSs isolated from WT, Δ*pde_pg_* and Δ*cdaR* mutants cultivated under low and high hemin conditions. **(A)** Endotoxin activities of various LPS samples (1 ng/ml). LPS samples derived from Δ*pde_pg_* and Δ*cdaR* mutants display significantly altered endotoxin activities relative to WT LPS. **(B)** The competitive or synergistic effects of LPS samples on *E*. *coli* LPS (LPS*_Ec_*; 0.5 Unit/ml) endotoxin activity. The presence of *P. gingivalis* WT and Δ*pde_pg_* LPS produced at low or high hemin conditions significantly exhibited synergistic effects, while the LPS sample of Δ*cdaR* mutant had no significant effect on the endotoxin activity of LPS*_Ec_*. The arrow indicates the baseline of the endotoxin activity of LPS*_Ec_*. **(C)** The immunostimulatory potential of LPSs was assessed using HEK-Blue™ hTLR4 (InvivoGen) reporter cells. In this assay, SEAP (secreted embryonic alkaline phosphatase) acts as a reporter for TLR4 ligand formation and activation. The graphs represent the mean ± SE of the endotoxin activity or immunostimulatory potential of each LPS sample (three biological replicates). Data were analyzed with a student’s *t-*test (* *p* < 0.05; **p < 0.01; **** *p <*0.0001; *ns* not significant). The standard curve of endotoxin activities is presented in **Supplementary Figure S11**.

The endotoxin activity of the LPS samples was determined using the clotting mechanism of the amebocyte lysate from the horseshoe crab (Limulus) (Tinker-Kulberg et al., 2020). As shown in **Figure 8**, LPS samples derived from the mutants displayed significantly altered endotoxin activities relative to WT LPS when grown in either low or high hemin conditions. Interestingly, the LPS of Δ*pde_pg_* and Δ*cdaR* mutants exhibited a reciprocal relationship, with Δ*pde_pg_* LPS showing a significant increase and Δ*cdaR* LPS a significant decrease in endotoxin activities compared to WT LPS. These differences were more pronounced in LPS produced at high hemin conditions. The activities of Δ*pde_pg_* and WT LPS molecules increased by approximately 7- and 10-fold, respectively, when compared to their counterparts produced at low hemin conditions. However, the activities of Δ*cdaR* LPS samples remained similar for both hemin conditions (**Figure 8A**).

Next, we aimed to determine the competitive or synergistic effects of *P. gingivalis* LPS molecules on *E. coli* LPS endotoxin activity. The presence of *P. gingivalis* WT and Δ*pde_pg_* LPS produced at low or high hemin conditions significantly augmented basal *E. coli* endotoxin activity, suggesting a synergistic effect (**Figure 8B**). LPS macromolecules from high hemin condition exhibited higher endotoxin activities compared to their cognate counterparts in low hemin condition. In contrast, the LPS samples of Δ*cdaR* mutant had no significant effect on *E. coli* LPS activity regardless of hemin concentration (**Figure 8B**). Collectively, these results strongly suggest that the function of CdaR is not only critical for c-di-AMP signaling but also plays a crucial role in determining LPS endotoxin activity.

The lipid A component of LPS primarily mediates the activation of the MD2/TLR4 signaling pathway in the innate immune system (Akashi et al., 2003; Park and Lee, 2013; Ciesielska et al., 2021). We employed HEK-Blue™ hTLR4 (InvivoGen) reporter cells to assess the effects of LPSs from the WT and mutant strains on TLR4 activation. In this assay, SEAP (secreted embryonic alkaline phosphatase) acts as a reporter for TLR4 ligand formation and activation. Our data revealed that LPS samples from WT cells grown under both low and high hemin conditions exhibited similar activities in inducing TLR4-dependent SEAP production (**Figure 8C**). However, the LPS from Δ*pde_pg_* and Δ*cdaR* cells grown at low and high hemin concentrations showed opposite activities. While TLR4 activation was highest for Δ*cdaR* LPS at low hemin condition compared to that of WT, Δ*pde_pg_* LPS exhibited a significantly higher potency of TLR4 activation than either the WT or Δ*cdaR* LPS samples at high hemin. These responses were TLR4-specific as HEK-293 Null Blue cells did not display any responses to the LPS molecules. Overall, these shifts in the biological activities and immunostimulatory potentials of the mutant LPS molecules signify significant changes in the chemical composition and/or relative abundance of different LPS variants within the same strain or mutant upon shifts in c-di-AMP signaling.

Given that finely tuned synthesis of c-di-AMP is required for the utilization and metabolic incorporation of exogenous pyruvate by *P. gingivalis* (Moradali and Davey, 2021; Moradali et al., 2022), we assessed the impact of pyruvate on the biological activities and immunostimulatory potential of the WT strain, correlating these findings with the cellular bioenergetic status and LPS compositional changes. As shown in **Figure 5**, and discussed earlier, the utilization of exogenous pyruvate by *P. gingivalis* resulted in an almost 3.3-fold increase in the percentage of total carbohydrates, a doubling of glucose content, as well as *Iso*-3-hydroxypentadecanoic acid [i15:0(3OH)]. LPS obtained from the pyruvate-treated cells displayed significantly higher immunoreactivity to the anti-*P. gingivalis* LPS antibody, as well as increased immunostimulatory activity in inducing TLR4-dependent SEAP production (**Supplementary Figure 10**). Interestingly, pyruvate supplementation led to a significant reduction in endotoxin activity compared to the non-treated condition in the LPS of the WT strain (**Supplementary Figure 10**). These data indicate that the metabolic and bioenergetic status of this anaerobe plays a significant role in determining the biological activities of LPS, as endotoxin activity is inversely correlated with the total carbohydrate percentage and glucose content.

## 4 Discussion

**Figure 9:**
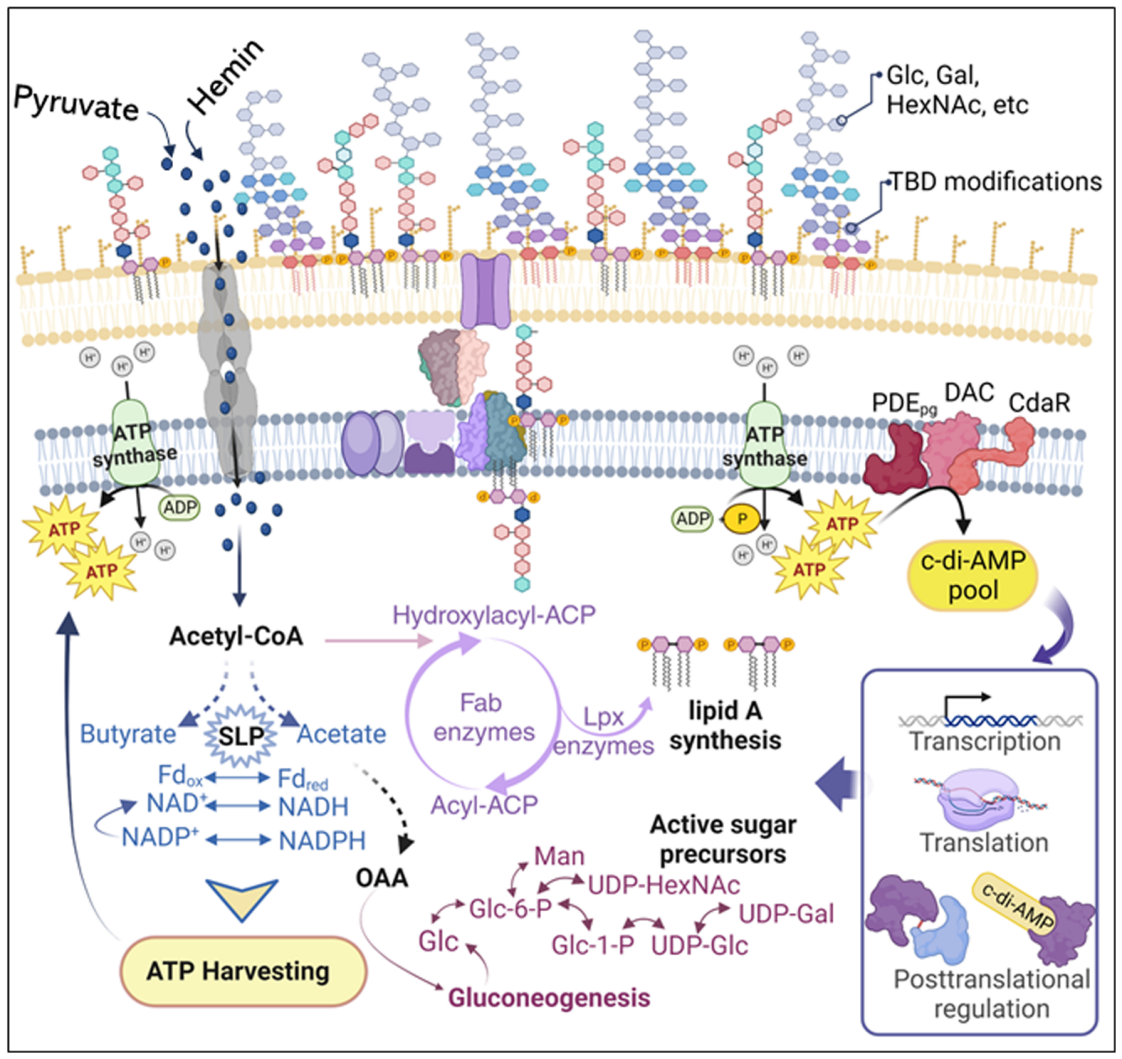
Schematic model illustrating the proposed mechanism of c-di-AMP regulation of *P. gingivalis* LPS biosynthesis and modifications. Changes in pyruvate or hemin availability occur in the subgingival niche during chronic infection and inflammation. *P. gingivalis* efficiently transports pyruvate and hemin, incorporating them into its metabolism to enhance ATP production and gluconeogenesis. Our previous studies (Moradali et al., 2019; Moradali and Davey, 2021) highlight that *P. gingivalis*’s bioenergetic status is primarily determined by coupling fermentative processes, redox reactions (NAD^+^/NADH; Fd_ox_/Fd_red_), substrate-level phosphorylation (e.g., conversion of acetyl-P to acetate and butyryl-P to butyrate), and concurrently elevating ATP generation. Gluconeogenesis, the *de novo* synthesis of glucose from non-hexose precursors, is crucial for biosynthetic processes, including the synthesis of fatty acid chains and glycosyl components of LPS macromolecules. Generally, gluconeogenesis correlates positively with cellular concentrations of ATP and acyl CoA, generating electron carriers (e.g., NADPH) and biosynthetic building blocks. Although anaerobic and asaccharolytic bacteria like *P. gingivalis* may differ in energy generation principles, our findings indicate that c-di-AMP signaling is essential for regulating bioenergetic status, concurrently influencing the biosynthesis of fatty acids and glycosyl compounds required for LPS biosynthesis. Our model proposes that the increase in cellular ATP concentration elevates the c-di-AMP pool, subsequently controlling desired biological outputs, including the LPS profile, at transcriptional, translational, or posttranslational levels. ATP, adenosine triphosphate; ADP, adenosine diphosphate; NAD nicotinamide adenine dinucleotide; NADPH, nicotinamide adenine dinucleotide phosphate; Fd_ox_, oxidized ferredoxin; Fd_red_, reduced ferredoxin; Glc, glucose; Glc-6-P, glucose-6-phosphate; Gal, galactose; Gal-6-P, galactose-6-phosphate; Man, mannose; HexNAc, N-acetylhexosamine; UDP, uridine diphosphate; OAA, oxaloacetate; CoA, coenzyme A; ACP, the acyl carrier protein; SLP, substrate-level phosphorylation; TBD, to be determined.

Our work provides some insights into the functional association between c-di-AMP signaling and the regulation of *P. gingivalis* LPS profile in response to changes in bioenergetic status. Pathogens employ a myriad of strategies to adapt to and persist within the host environment, evading defense mechanisms to promote chronic infections (Cullen and Mcclean, 2015; Maldonado et al., 2016; Sengupta et al., 2016; Moradali et al., 2017). Hence, the dynamic regulation of LPS profiles emerges as a crucial strategy for the virulence community of Gram-negative bacteria, inducing the development of chronic periodontitis (Millar et al., 1986; Bainbridge and Darveau, 2001; Darveau et al., 2002; Dixon and Darveau, 2005). While previous studies have substantially contributed to delineating variations in *P. gingivalis* LPS profiles, how these variations arise has not been thoroughly investigated. Indeed, the biosynthesis of LPS is highly complex, requiring the engagement of multiple biosynthetic pathways and the involvement of multi-level regulatory mechanisms (Moradali and Rehm, 2020; Swietnicki and Caspi, 2021). Thus, our study reinforces the significance of employing a comprehensive technical approach to decipher the functional relationship between c-di-AMP signaling and the complex LPS structure in *P. gingivalis*. The results presented in this study establish a critical link between c-di-AMP signaling, cellular bioenergetics, and the regulation of *P. gingivalis* LPS profile. A schematic model illustrating the proposed mechanism of c-di-AMP regulation of *P. gingivalis* LPS biosynthesis and modifications is presented in **Figure 9**. Our investigation stems from the observation that mutants Δ*pde_pg_* and Δ*cdaR*, with dysregulated c-di-AMP regulation, not only exhibit aberrations in ATP levels which is key to cellular bioenergetics, but also display significant alterations in the LPS-driven phenotype (Moradali et al., 2022). Our findings demonstrate that pyruvate and hemin profoundly influence the bioenergetic state of the bacterium, as well as the LPS profile. This influence extends to the alteration of cellular ATP and consequently c-di-AMP levels, aligning with our earlier observations regarding the utilization of exogenous pyruvate by *P. gingivalis* (Moradali and Davey, 2021) (**Figure 1**). Consistent with our prior study (Moradali et al., 2022), our current findings highlight an atypical regulatory mechanism governing cellular c-di-AMP levels in *P. gingivalis*, and potentially in other Bacteroidota. Unlike the typical scheme observed mainly in Gram-positive species, where c-di-AMP synthesis and its degradation by specific phosphodiesterases regulate potassium transporter systems, osmotic homeostasis, or other targets (Krüger et al., 2021; Schwedt et al., 2023), our data demonstrate PDE_pg_ does not act as a typical phosphodiesterase in *P. gingivalis*. Instead, both PDE_pg_ and CdaR play an indispensable role in facilitating the incorporation of ATP into the c-di-AMP molecule, particularly under permissive conditions conducive to alterations in cellular ATP levels (Moradali et al., 2022). This underscores the regulatory complexity within such a slow-growing strict anaerobe in response to changes in cellular bioenergetic status. As revealed in this study, the intricate balance between c-di-AMP signaling and ATP-driven cellular bioenergetics sets the stage for governing stimulus-induced variations in *P. gingivalis* LPS, while excluding global lipid alterations. Moreover, our investigation, exploring beyond lipid A analysis, revealed intricate shifts in fatty acid and glycosyl profiles upon c-di-AMP dysregulation. We posit that c-di-AMP functions as a metabolic nexus, linking the metabolic status to the nuanced regulation of specific fatty acid and glycosyl residues. These include various isoforms of glucose, galactose, rhamnose, and specific amino sugars. Notably, the absence, or significant reduction, of N-acetyl galactosamine upon c-di-AMP dysregulation further highlights the regulatory role of this signaling system in shaping the chemical composition of LPS and its connection to central metabolism. This is further supported by the SDS-PAGE-based approach, which shows that CdaR activity is crucial for producing band 1, a novel positively charged LPS-associated glycosyl variant in *P. gingivalis* LPS profile. While our preliminary analysis could not pinpoint the exact composition of band 1, our analytical data collectively suggest the presence of glucose and amino sugar components in this band. This potential connection between the absence of N-acetyl galactosamine and the absence of band 1 in the Δ*cdaR* mutant, and the growth defect of this mutant (Moradali et al., 2022) awaits further investigation to unravel their underlying molecular interactions. Furthermore, *cdaR* deletion imposes profound effects on the integrity of *P. gingivalis* cell envelope (Moradali et al., 2022) and the lipid A profile. This implies that CdaR`s regulatory function may not be limited to the LPS profile alone but may have pleiotropic effects on multiple biosynthesis pathways as well. Additionally, the low abundance of the pentaacyl-P1 (III) variants in Δ*cdaR* mutant under both hemin conditions, along with the absence of band 1 in this mutant, underscores the complexity of LPS biosynthesis and regulation in *P. gingivalis*, warranting further investigation. Previous studies have shown that hemin-dependent lipid A phosphatases regulate lipid A chemical composition and the relative abundance of lipid A variants with varying degrees of phosphorylation and acylation within the same strain (Coats et al., 2009). However, the mechanism through which c-di-AMP signaling potentially affects the regulation of these phosphatases at transcriptional or post-translational levels remain to be explored.

Signaling via second messengers, such as c-di-AMP or cyclic di-3′, 5′-guanylic acid (c-di-GMP), acts as a molecular orchestrator, integrating stimuli and cellular processes to promote microbial adaptation (Foreman-Wykert and Miller, 2003; Choi and Groisman, 2013; Pontes et al., 2015; Moradali et al., 2017; Olsen and Singhrao, 2018). C-di-AMP is generally known as an ‘essential poison’ (Gundlach et al., 2015; Commichau et al., 2019) which has the potential to impose toxic effects on bacterial growth. We do not yet understand the possible pleiotropic effects of changes in cellular c-di-AMP concentration on *P. gingivalis* physiology. Given the involvement of multiple cellular pathways in the complex LPS biosynthesis, and the discernible impact of c-di-AMP signaling on various components of *P. gingivalis* LPS, our data underscore the multifaceted role of c-di-AMP signaling in *P. gingivalis* physiology (**Figure 9**). This aligns with emerging literature on the diverse roles of c-di-AMP signaling in bacterial physiology (Römling, 2008; Corrigan et al., 2011; Oppenheimer-Shaanan et al., 2011; Luo and Helmann, 2012; Corrigan and Gründling, 2013; Corrigan et al., 2013; Peng et al., 2016). The pathoadaptive mechanism of c-di-AMP-regulated LPS structure and function in *P. gingivalis* is analogous to the control of the LPS profile by c-di-GMP signaling in *Salmonella typhimurium* and *Pseudomonas aeruginosa* (Foreman-Wykert and Miller, 2003; Choi and Groisman, 2013; Pontes et al., 2015; Moradali et al., 2017; Olsen and Singhrao, 2018). Additionally, similar structural rearrangements of the entire LPS macromolecules in *Helicobacter pylori* underlie the LPS-driven “persistence mechanisms” of the pathogen (Chmiela et al., 2014). These mechanisms allow to colonize the mucus layer, modulate immune responses, and promote persistence in the human host (Chmiela et al., 2014). On the other hand, community-wide genomic and transcriptomic analyses have revealed the highly active biosynthesis of LPS components in Gram-negative bacteria, along with components of c-di-AMP signaling in the periodontal pockets of subjects with periodontitis (Yost et al., 2015; Solbiati and Frias-Lopez, 2018; Ram-Mohan and Meyer, 2020). Hence, this newfound understanding not only extends the frontiers of our comprehension of c-di-AMP signaling pathways in Gram-negative species but also lays a foundation for unraveling the mechanistic basis of variations in the LPS profile and its contribution to disease progression. The profound impact of c-di-AMP signaling on modulating the biological activities and immunostimulatory potential of LPSs, as identified in our study, underscores the significance of this signaling system in host-pathogen interactions and immune evasion in the context of chronic infections. Moreover, our study highlights the specific roles of different LPS constituents in the pathogen’s interaction with various primary defense mechanisms, as previously addressed (Al-Qutub et al., 2006; Coats et al., 2009; Curtis et al., 2011; Herath et al., 2013; Zaric et al., 2017; Olsen and Singhrao, 2018). We observed the necessity of SDS-PAGE-based normalization, using lipid A band intensity as a reference for applied LPS samples before biological assays, as application by weight is inaccurate and does not account for possible unknown components or impurities. This issue was also noted with commercial products of *P. gingivalis* LPS, which exhibited significant impurities and unassignable *m/z* values. Given the near absence of endotoxin activity in Δ*cdaR* LPS and the reliance of endotoxin activity on LPS attributes for micelle particle formation (Harm et al., 2021; Fux et al., 2023), it underscores the necessity to account for the impact of chemical modifications and variations on the biophysical characteristics of *P. gingivalis* LPS. This consideration extends beyond the mere ratio of different lipid A variants. However, our understanding of the exact chemical composition and modifications of *P. gingivalis* LPS components, such as lipid A/core oligosaccharide and A-type polysaccharide, highly impacted by c-di-AMP dysregulation, remains limited. Furthermore, it is still unclear whether elevated hemin concentration may affect osmolarity, subsequently influencing cellular processes and signaling systems necessary for adaptation to new conditions. Therefore, future work will aim to address these gaps. Moreover, our ongoing research aims to elucidate the mechanistic basis of c-di-AMP regulation of LPS structure and its contribution to disease progression in the periodontal microbiome. Overall, our data support the notion that c-di-AMP-regulated LPS structure, and consequently its function, serves as a key pathoadaptation mechanism for *P. gingivalis*, and potentially other Bacteroidota allowing selective modification of the LPS profile in response to shifts in bioenergetic status in the ever-changing environment of the oral cavity. These mechanisms are crucial for modulating interactions with the host and polymicrobial community, enabling pathogens to overcome multiple primary defense mechanisms and promote persistent infections. Understanding the significance of c-di-AMP-regulated LPS structure in pathoadaptation is not only crucial for deciphering the molecular mechanisms employed by pathogens during chronic infections but also opens avenues for targeted therapeutic interventions.

## Data availability statement

The original contributions presented in the study are included in the article/**Supplementary Material**. Further inquiries can be directed to the corresponding author.

## Author contributions

SG: Data curation, Formal analysis, Investigation, Methodology, Software, Validation, Visualization, Writing – original draft, Writing – review & editing. ArM: Data curation, Formal analysis, Methodology, Writing – review & editing. HY: Formal analysis, Writing – review & editing. RS: Formal analysis, Writing – review & editing. AsM: Formal analysis, Writing – review & editing. JH: Formal analysis, Writing – review & editing. GK: Formal analysis, Writing – review & editing. FN: Data curation, Formal analysis, Writing – review & editing. PA: Data curation, Formal analysis, Writing – review & editing, Funding acquisition, Methodology. RE: Data curation, Formal analysis, Funding acquisition, Methodology, Writing – review & editing. FM: Conceptualization, Data curation, Formal analysis, Funding acquisition, Methodology, Writing – review & editing, Project administration, Resources, Software, Supervision, Validation, Visualization, Writing – original draft.

## Supplementary material

Supplementary Figure 1 Lipidomic analysis of *P. gingivalis* strain 381 (WT), ∆*pde_pg_* and ∆*cdaR* mutants. **(A, B)** Common lipid classes in *P. gingivalis*, including dihydroceramides, phospholipids, and serine-glycine lipodipeptides. **(C–J)** Comparative statistical analysis of the amounts of different lipid classes identified in the lipidomic analysis of WT, ∆*pde_pg_*, and ∆*cdaR* mutants. This experiment was performed three times and graphs represent the mean ± SE of the identified lipids which were analyzed with a student’s *t*-test (ns, not significant). Raw data of each lipid class is presented in **Supplementary Table 1**. WT, wild type; H, hemin (1 or 10 µg/ml). PGDHC, phosphoglycerol dihydroceramide; PEDHC, phosphoethanolamine dihydroceramide; PEA, phosphoethanolamine; L430/576/654/ 1256/1242, lipodipeptides.

Supplementary Figure 2 Lipid A variants in the LPS profile of *P. gingivalis* identified through the FLAT^*n*^ analysis. **(A)** Predominant lipid A variants in *P. gingivalis* strain 381, and **(B)** the FLAT^*n*^ spectrum along with the ratio of the lipid A variants.

Supplementary Figure 3 FLAT^*n*^ mass spectra from different precursor ions and structure determination of different lipid A variants and list of assigned ions.

Supplementary Figure 4 Comparison of the FLAT^*n*^/LPS_pure_ **(A)** and the FLAT^*n*^/in situ **(B)** mass spectra. ND: not detected. H, hemin (1 or 10 µg/ml).

Supplementary Figure 5 Lipidomic analysis to assess the impact of utilization of exogenous pyruvate on different lipid classes of *P. gingivalis*. **(A–H)** Comparative statistical analysis of different lipid classes identified in the lipidomic analysis shows no significant difference between pyruvate supplementation and untreated conditions. Graphs represent the mean ± SE (three biological replicates) of the identified lipids which were analyzed with a student’s *t*-test (ns, not significant). WT, wild type; PGDHC, phosphoglycerol dihydroceramide; PEDHC, phosphoethanolamine dihydroceramide; PEA, phosphoethanolamine; L430/576/654/1256/1242, lipodipeptides.

Supplementary Figure 6 The major *m/z* signals greater than *m/z* 1871 observed in the FLAT^*n*^/LPS_pure_ analysis, with dissimilar intensity and relative abundance between WT and mutants (∆*pde_pg_* and ∆*cdaR*) under low and high hemin conditions. Asterisks indicate major *m/z* signals assignable to the lipid A/core oligosaccharide region that are absent or shifted in the LPS of the mutants compared to WT.

Supplementary Figure 7 Chromatograms of the trimethylsilyl-methyl glycosides and fatty acid residues detected in the LPS of WT, grown with or without pyruvate, and ∆*pde_pg_* and ∆*cdaR* mutants grown in low or high hemin concentrations **(A)** and SDS-PAGE analysis of LPS isolated from pyruvate-treated and non-treated cells **(B)**. Pro-Q Emerald 300 LPS kit was used to stain LPS bands. HMW, high molecular weight.

Supplementary Figure 8 DOC-PAGE profile of silver (left) and Alcian blue and silver stained (right) gels of the LPS of WT and mutants. 1 µg LPS was used in each lane. Lane (S): control LPS from *Salmonella enterica serovar Typhimurium*.

Supplementary Figure 9 The compositional analysis of band no. 1 extracted from SDS-PAGE. **(A)** MS/MS and FLAT^*n*^ analyses show several signals that result from consecutive loss of the *m/z* 287 ion, indicating the polymeric nature of this component. **(B)** GC-MS peaks demonstrated traces of hexoses, including glucose, amino sugars, and glycerol in band no. 1. No common LPS fatty acid residues were detected in the analyzed fraction.

Supplementary Figure 10 Impact of pyruvate on the biological activity and immunostimulatory potential of *P. gingivalis* LPS. **(A)** ELISA assay was performed using anti-*P. gingivalis* LPS monoclonal antibody. LPS obtained from the pyruvate-treated cells displayed significantly higher immunoreactivity to the anti-*P. gingivalis* LPS antibody. **(B)** HEK-Blue^TM^ hTLR4-based assay. LPS obtained from the pyruvate-treated cells exhibits increased immunostimulatory activity in inducing TLR4-dependent SEAP production. **(C)** Endotoxin activity. Although WT LPS from the low hemin condition exhibits very low endotoxin activity, pyruvate supplementation led to a significant reduction in endotoxin activity compared to the non-treated condition in the LPS of the WT strain. The graph represents the mean ± SE of the immunoreactivity of each LPS sample (three biological replicates). Data were analyzed with a student’s *t*-test (**** *p* <0.0001).

Supplementary Figure 11 Standard curves used in this study. **(A)** ATP quantification assay. **(B)** C-di-AMP quantification assay. **(C)** Endotoxin activity assay.
