# Supplementary material for: The multifaceted role of c-di-AMP signaling in the regulation of Porphyromonas gingivalis lipopolysaccharide structure and function
Source: Front Cell Infect Microbiol. 2024 Jun 12;14:1418651. doi: 10.3389/fcimb.2024.1418651 (PMC11199400; doi:10.3389/fcimb.2024.1418651)
Supplement: Supplementary Figure 1 — Lipidomic analysis of P. gingivalis strain 381 (WT), ∆pdepg and ∆cdaR mutants. (A, B) Common lipid classes in P. gingivalis, including dihydroceramides, phospholipids, and serine-glycine lipodipeptides. (C–J) Comparative statistical analysis of the amounts of different lipid classes identified in the lipidomic analysis of WT, ∆pdepg , and ∆cdaR mutants. This experiment was performed three times and graphs represent the mean ± SE of the identified lipids which were analyzed with a student’s t-test (ns, not significant). Raw data of each lipid class is presented in Supplementary Table 1 . WT, wild type; H, hemin (1 or 10 µg/ml). PGDHC, phosphoglycerol dihydroceramide; PEDHC, phosphoethanolamine dihydroceramide; PEA, phosphoethanolamine; L430/576/654/ 1256/1242, lipodipeptides. [file DataSheet_1.pdf]

Fig. S1

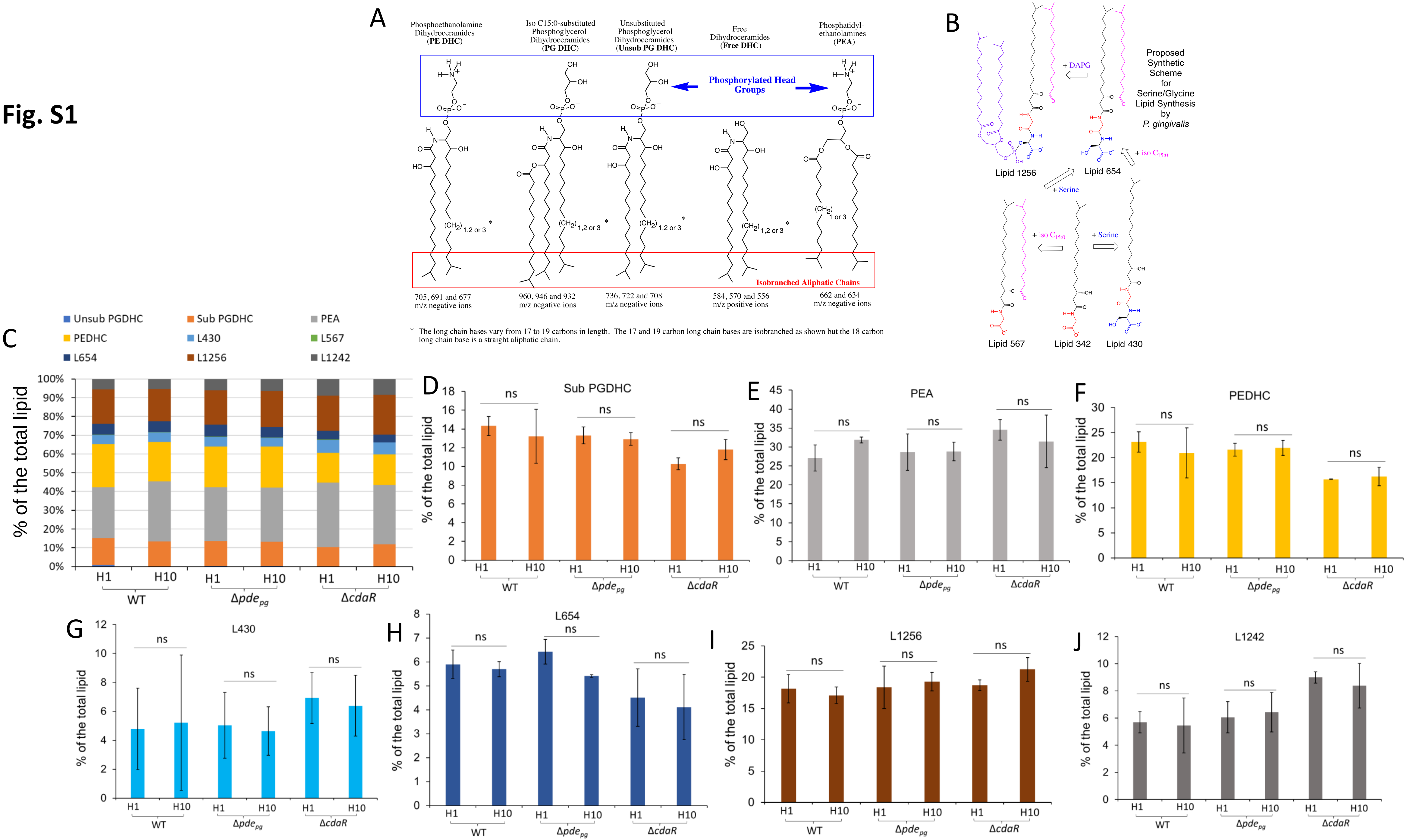
A
Phosphoethanolamine Dihydroceramides (PE DHC)
Iso C15:0-substituted Phosphoglycerol Dihydroceramides (PG DHC)
Unsubstituted Phosphoglycerol Dihydroceramides (Unsub PG DHC)
Free Dihydroceramides (Free DHC)
Phosphatidyl-ethanolamines (PEA)
Phosphorylated Head Groups
Isobranched Aliphatic Chains
705, 691 and 677 m/z negative ions
960, 946 and 932 m/z negative ions
736, 722 and 708 m/z negative ions
584, 570 and 556 m/z positive ions
662 and 634 m/z negative ions
* The long chain bases vary from 17 to 19 carbons in length. The 17 and 19 carbon long chain bases are isobranched as shown but the 18 carbon long chain base is a straight aliphatic chain.
B
Proposed Synthetic Scheme for Serine/Glycine Lipid Synthesis by P. gingivalis
+ DAPG
+ iso C15:0
+ Serine
Lipid 1256
Lipid 654
Lipid 567
Lipid 342
Lipid 430
C
Unsub PGDHC
Sub PGDHC
PEA
PEDHC
L430
L567
L654
L1256
L1242
% of the total lipid
H1
H10
WT
Δpde_pg
ΔcdaR
D Sub PGDHC
E PEA
F PEDHC
G L430
H L654
I L1256
J L1242
ns

I
m/z 1434

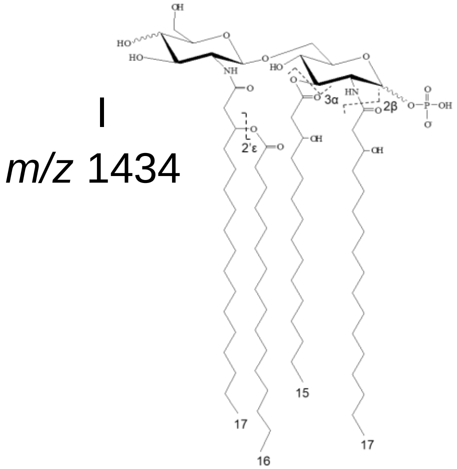

II
m/z 1448

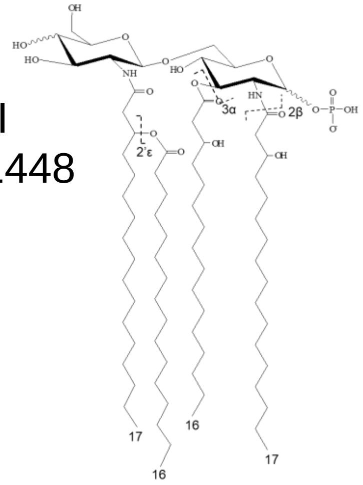

III
m/z 1688
m/z 1702

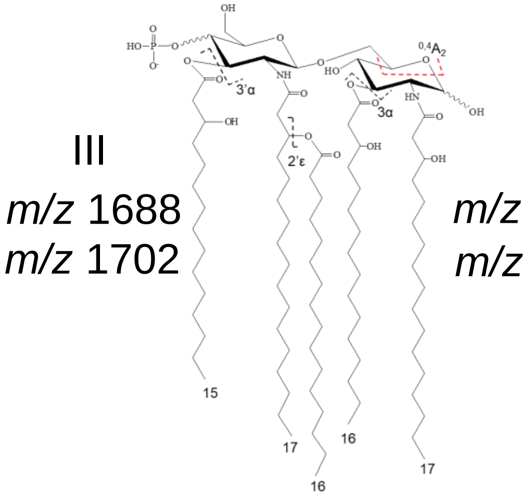

IV
m/z 1768
m/z 1782

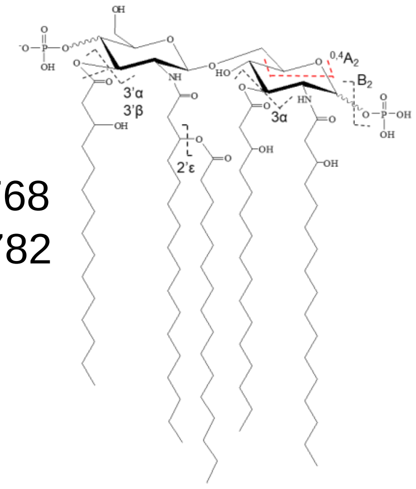

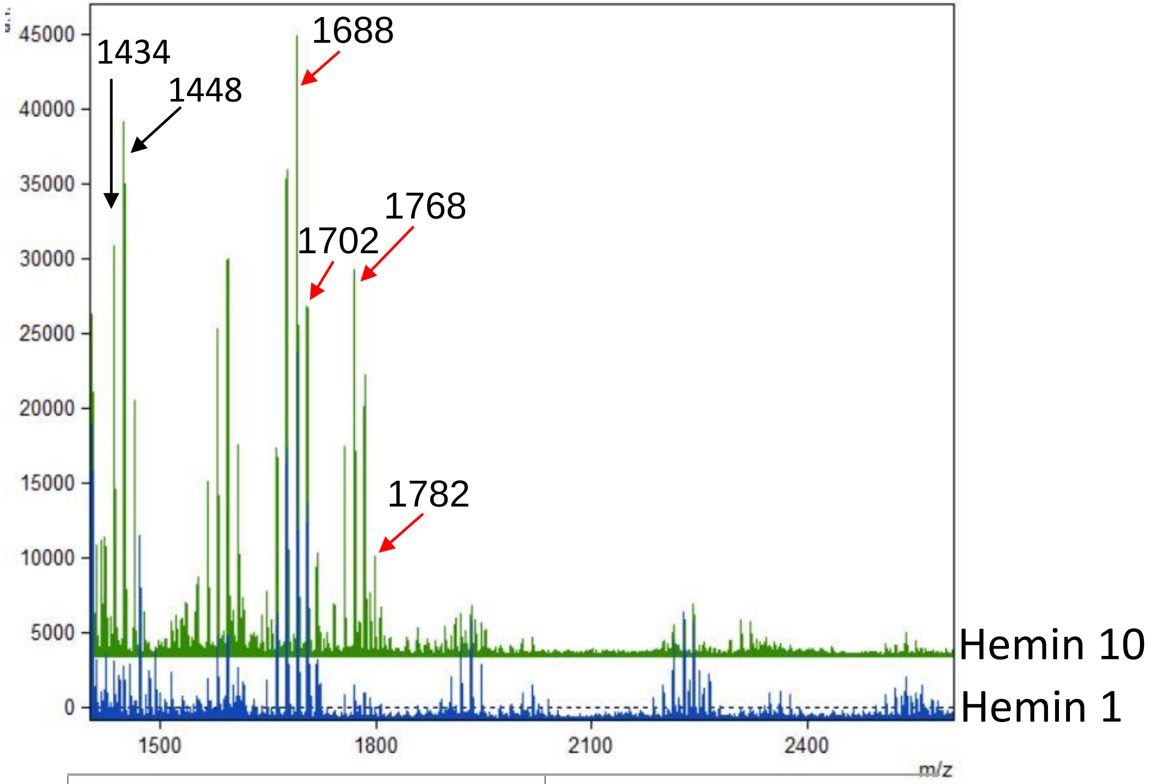

	H1	H 10
$I_{1448}/(I_{1448}+I_{1782})$	n/a	0.64
$I_{1448}/(I_{1448}+I_{1768})$	0.65	0.57
$I_{1448}/(I_{1448}+I_{1702})$	0.28	0.58
$I_{1448}/(I_{1448}+I_{1688})$	0.19	0.45

Fig. S2

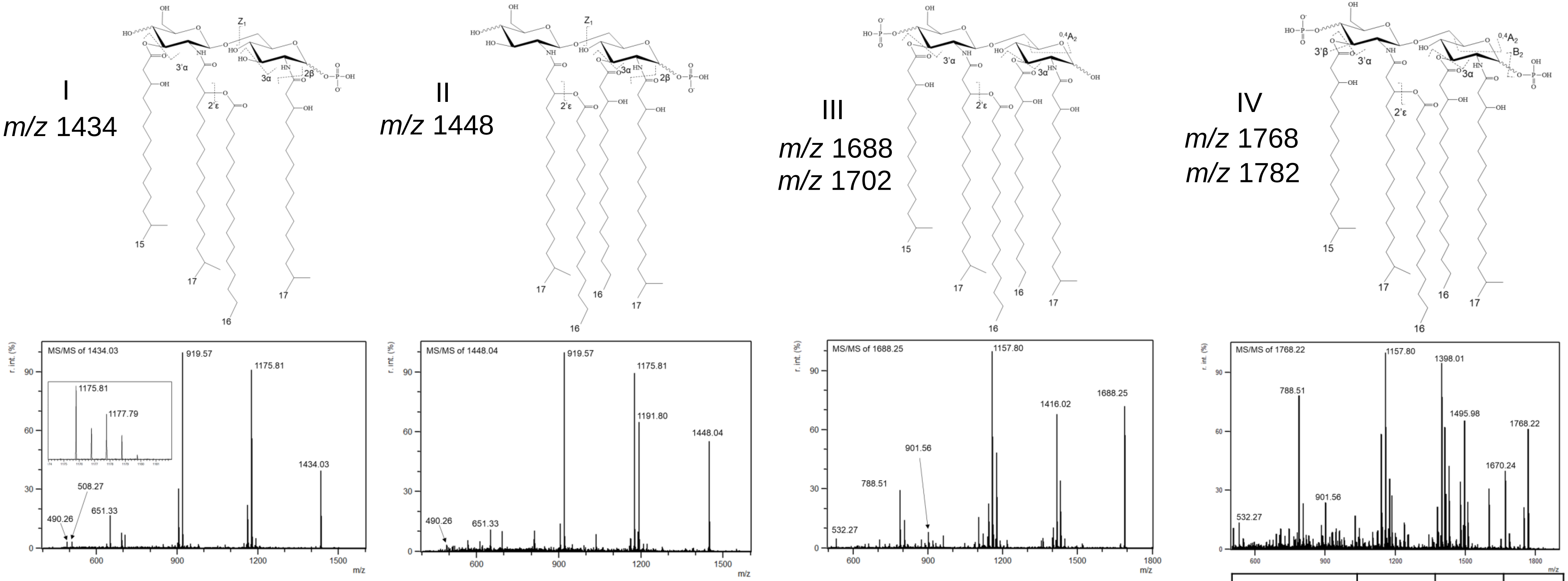

Naming	*m/z* (theory)	*m/z* (expt)	Error (Da)
3'α	1175.81	1175.79	0.02
2'ε	1177.79	1177.77	0.02
3'α + 2'ε	919.57	919.55	0.02
3'α + 2'ε + 2β	651.33	651.31	0.02
Z_1	508.27	508.26	0.01
Z_1 + 3α	490.26	490.25	0.01

Naming	*m/z* (theory)	*m/z* (expt)	Error (Da)
3α	1175.81	1175.80	0.01
2'ε	1191.80	1177.80	0.00
3'α + 2'ε	919.57	919.57	0.00
3'α + 2'ε + 2β	651.33	651.33	0.00
Z_1 + 3α	490.26	490.26	0.00

Naming	*m/z* (theory)	*m/z* (expt)	Error (Da)
3α	1416.02	1415.99	0.03
3α + 3'α	1157.80	1157.78	0.02
3α + 3'α + 2'ε	901.56	901.54	0.02
3'α + $^{0,4}A_2$	788.51	788.50	0.01
3'α + 2'ε + $^{0,4}A_2$	532.27	532.26	0.01

Naming	*m/z* (theory)	*m/z* (expt)	Error (Da)
B_2	1670.24	1670.24	0.00
3α	1495.98	1495.98	0.00
B_2 + 3α	1398.01	1398.01	0.00
$B_2 + 3^{α}$ + 3'β	1157.80	1157.80	0.00
$B_2 + 3^{α}$ + 3'β + 2'ε	901.56	901.56	0.00
3'α + $^{0,4}A_2$	788.51	788.50	0.01
3'α + 2'ε + $^{0,4}A_2$	532.27	532.27	0.00

Fig. S3

The FLAT^n/LPS$_{pure}$

The FLAT^n/*in situ*

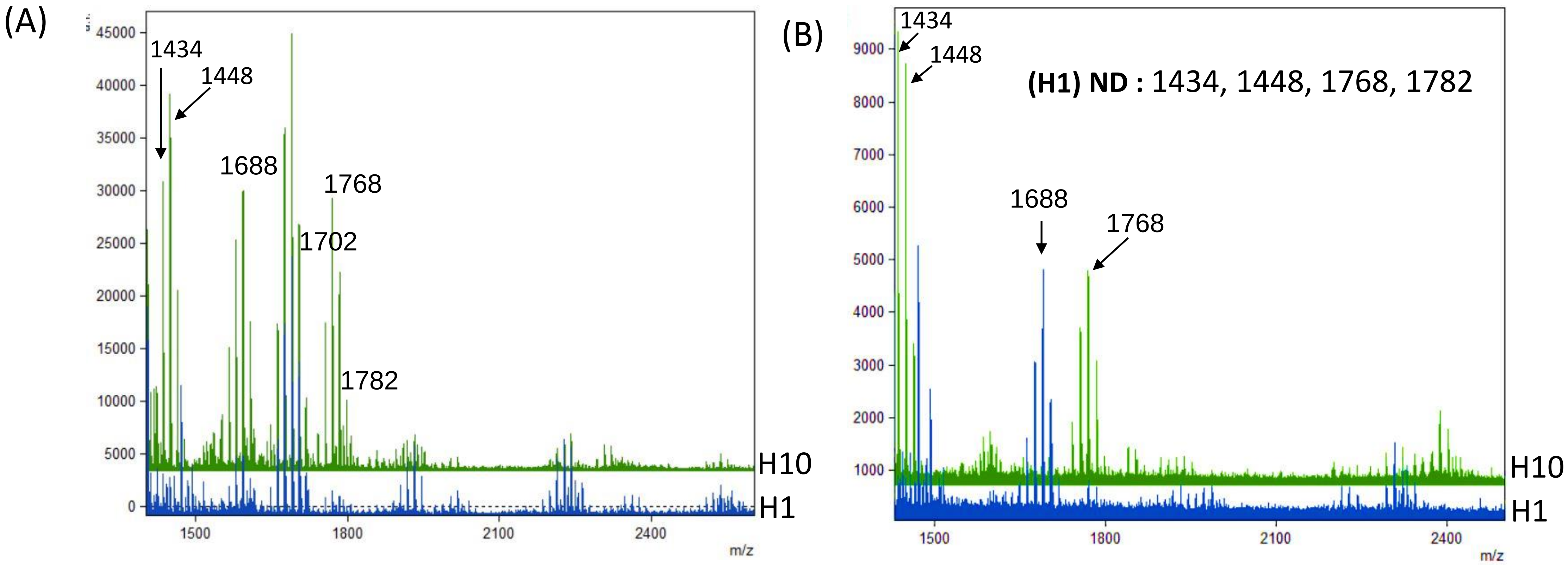

Fig. S4

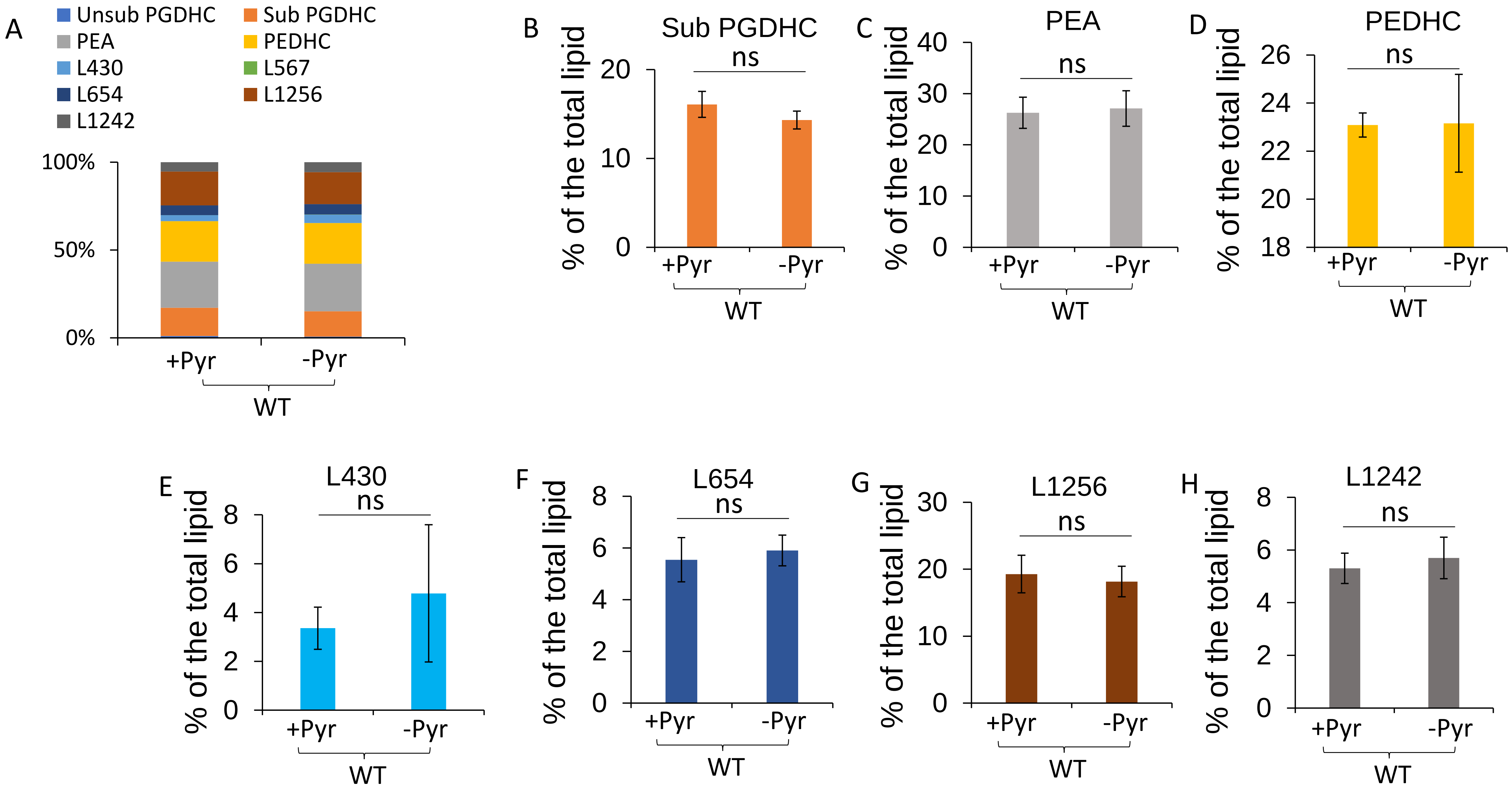
A
Unsub PGDHC
Sub PGDHC
PEA
PEDHC
L430
L567
L654
L1256
L1242
100%
50%
0%
+Pyr
-Pyr
WT
B
Sub PGDHC
ns
% of the total lipid
20
10
0
+Pyr
-Pyr
WT
C
PEA
ns
% of the total lipid
40
30
20
10
0
+Pyr
-Pyr
WT
D
PEDHC
ns
% of the total lipid
26
24
22
20
18
+Pyr
-Pyr
WT
E
L430
ns
% of the total lipid
8
6
4
2
0
+Pyr
-Pyr
WT
F
L654
ns
% of the total lipid
8
6
4
2
0
+Pyr
-Pyr
WT
G
L1256
ns
% of the total lipid
30
20
10
0
+Pyr
-Pyr
WT
H
L1242
ns
% of the total lipid
8
6
4
2
0
+Pyr
-Pyr
WT

Fig. S5

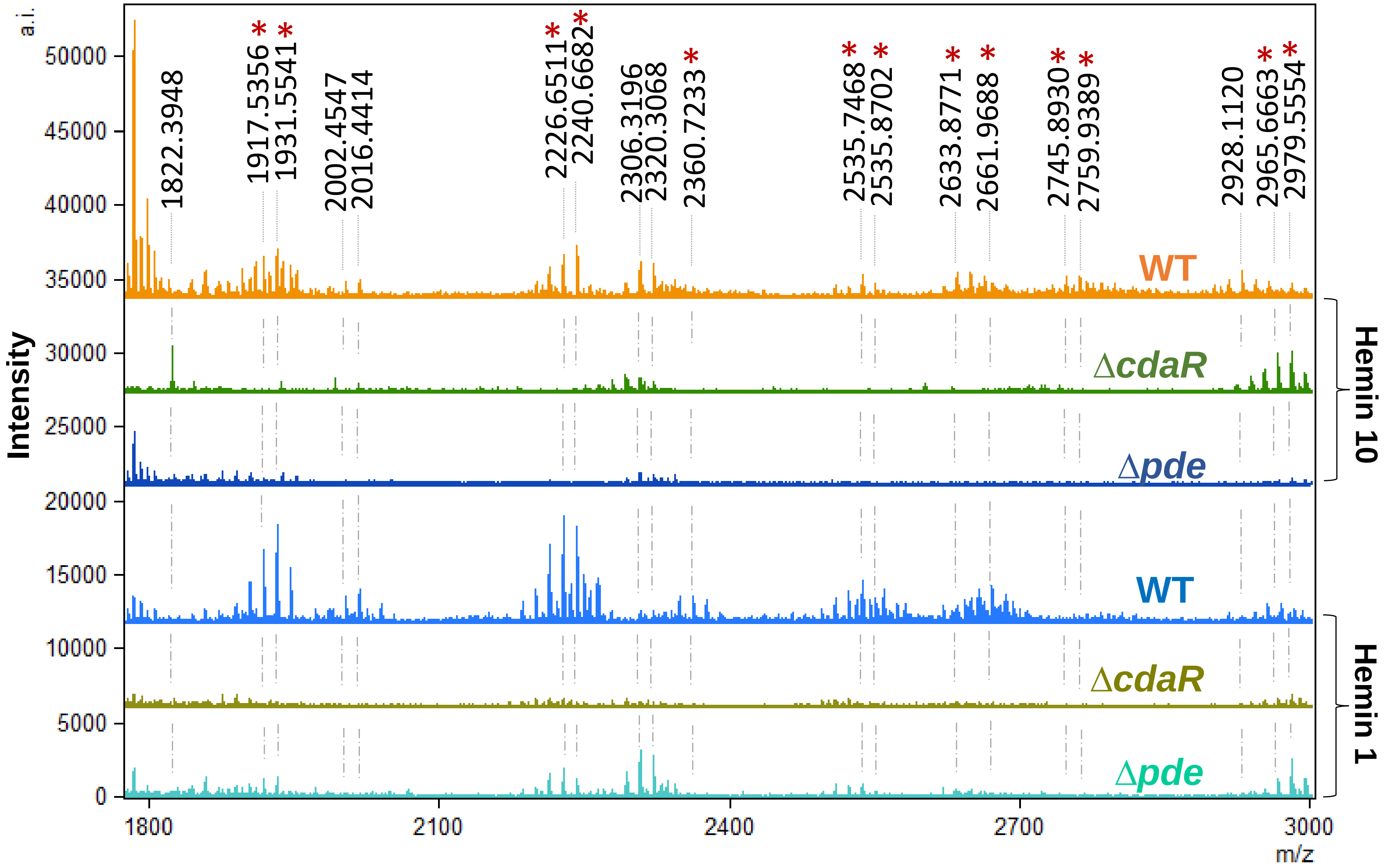
a.i.
Intensity
50000
45000
40000
35000
30000
25000
20000
15000
10000
5000
0
1822.3948
1917.5356 *
1931.5541 *
2002.4547
2016.4414
2226.6511 *
2240.6682 *
2306.3196
2320.3068
2360.7233 *
2535.7468 *
2535.8702 *
2633.8771 *
2661.9688 *
2745.8930 *
2759.9389 *
2928.1120
2965.6663 *
2979.5554 *
WT
ΔcdaR
Δpde
WT
ΔcdaR
Δpde
Hemin 10
Hemin 1
1800
2100
2400
2700
3000
m/z

Fig. S6

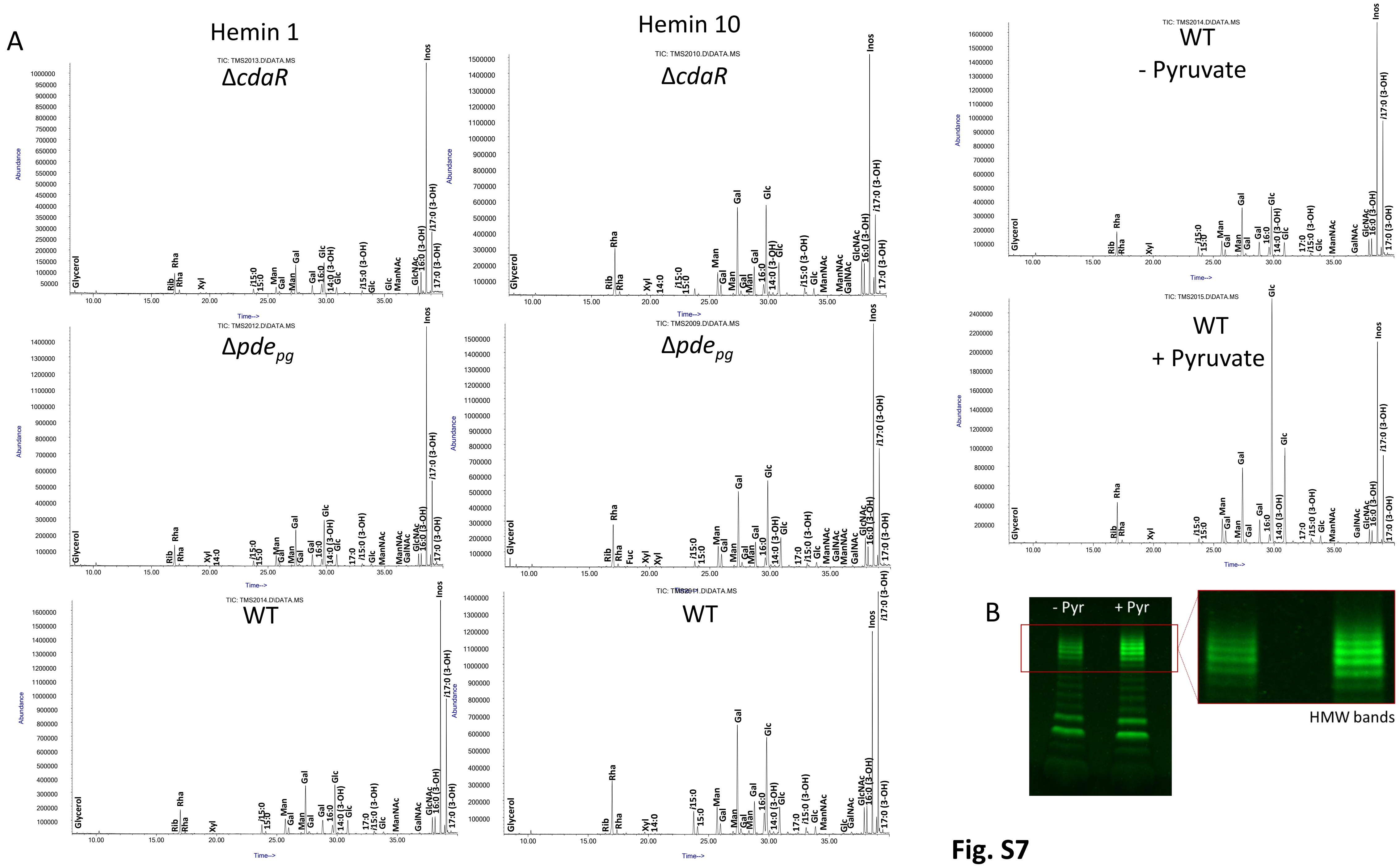
A
Hemin 1
Hemin 10
TIC: TMS2013.D\DATA.MS
ΔcdaR
TIC: TMS2010.D\DATA.MS
ΔcdaR
TIC: TMS2014.D\DATA.MS
WT
- Pyruvate
TIC: TMS2012.D\DATA.MS
Δpde_pg
TIC: TMS2009.D\DATA.MS
Δpde_pg
TIC: TMS2015.D\DATA.MS
WT
+ Pyruvate
TIC: TMS2014.D\DATA.MS
WT
WT
Abundance
Time-->
Glycerol
Rib
Rha
Xyl
Fuc
14:0
i15:0
15:0
Man
Gal
Glc
16:0
14:0 (3-OH)
i15:0 (3-OH)
17:0
ManNAc
GalNAc
GlcNAc
16:0 (3-OH)
Inos
i17:0 (3-OH)
17:0 (3-OH)
B
- Pyr
+ Pyr
HMW bands

Fig. S7

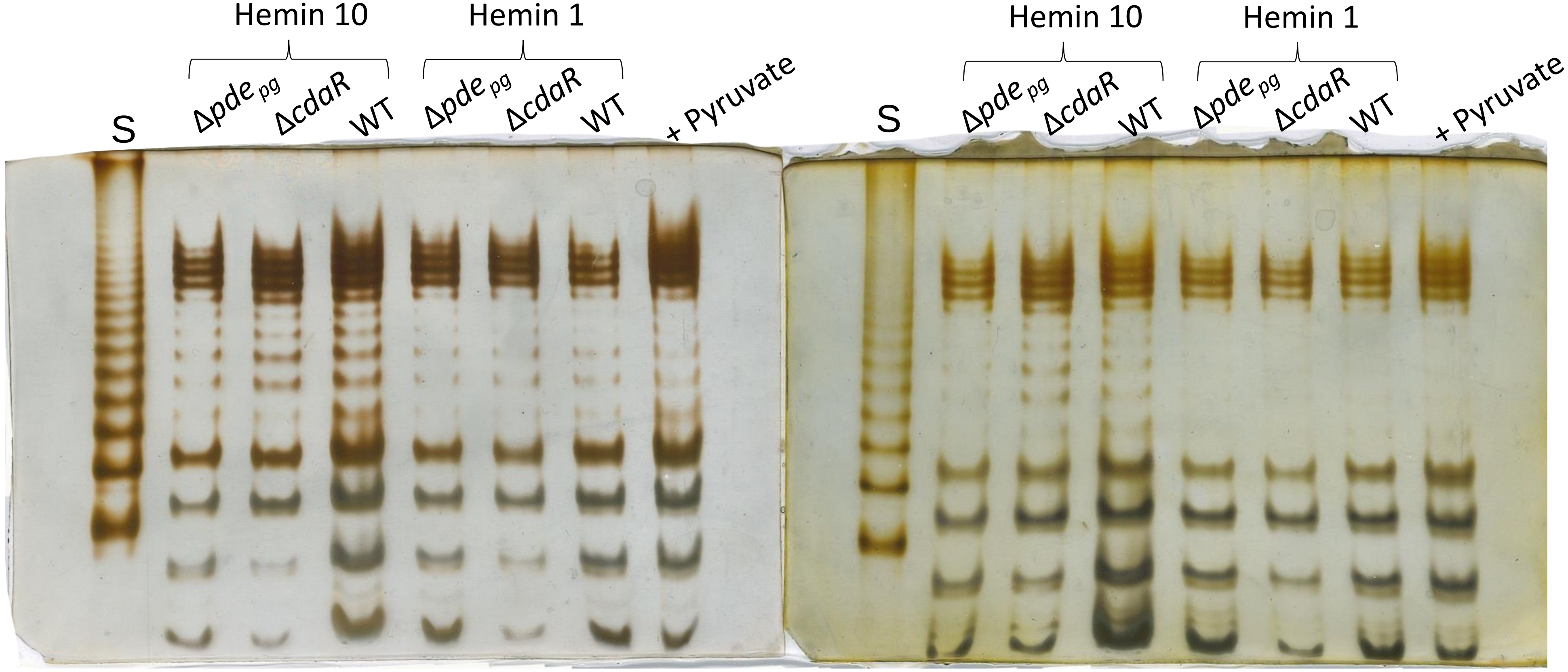
Hemin 10
Hemin 1
S
Δpde pg
ΔcdaR
WT
Δpde pg
ΔcdaR
WT
+ Pyruvate
Hemin 10
Hemin 1
S
Δpde pg
ΔcdaR
WT
Δpde pg
ΔcdaR
WT
+ Pyruvate

Fig. S8

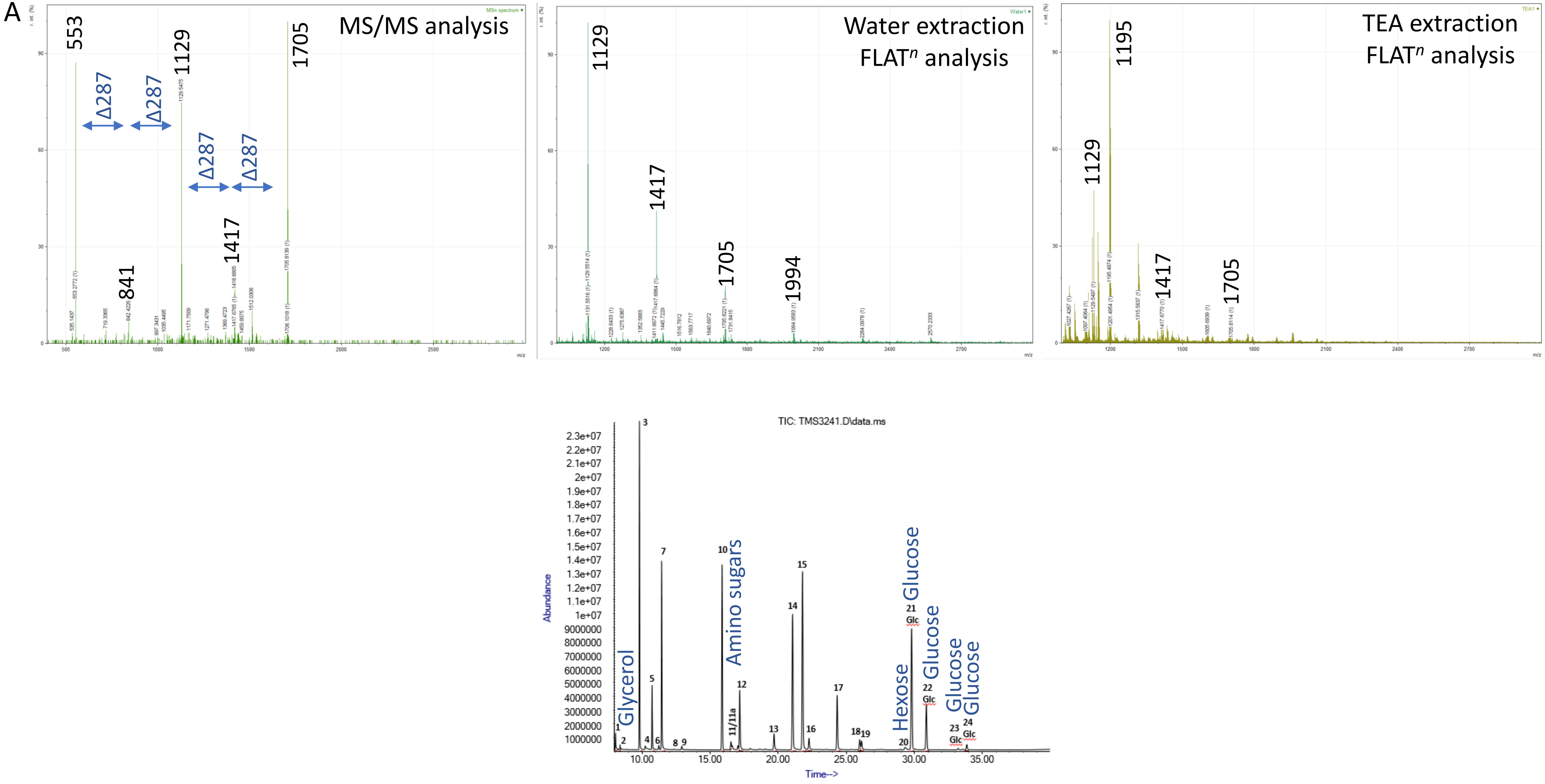
A
MS/MS analysis
553
Δ287
Δ287
1129
Δ287
Δ287
1705
841
1417
Water extraction
FLAT^n analysis
1129
1417
1705
1994
TEA extraction
FLAT^n analysis
1195
1129
1417
1705
TIC: TMS3241.D\data.ms
Abundance
Time-->
Glycerol
Amino sugars
Hexose
Glucose
Glucose
Glucose
Glucose

Fig. S9

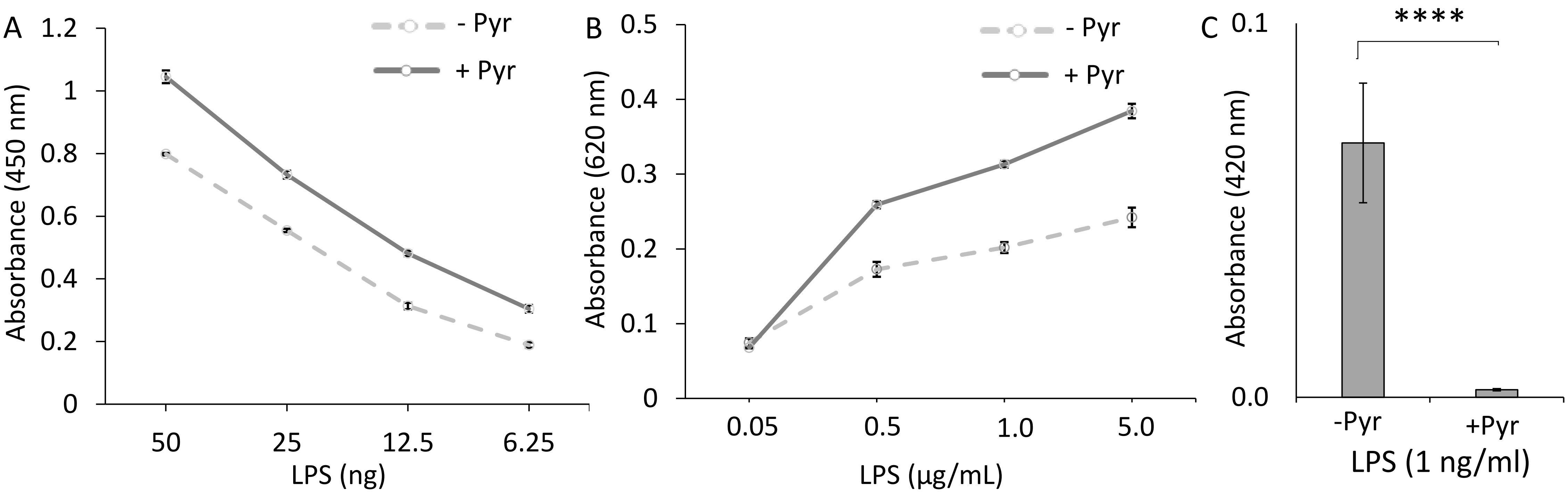
A
B
C
- Pyr
+ Pyr
Absorbance (450 nm)
1.2
1
0.8
0.6
0.4
0.2
0
50
25
12.5
6.25
LPS (ng)
Absorbance (620 nm)
0.5
0.4
0.3
0.2
0.1
0
0.05
0.5
1.0
5.0
LPS (µg/mL)
****
Absorbance (420 nm)
0.1
0.0
-Pyr
+Pyr
LPS (1 ng/ml)

Fig. S10

Standard Curves

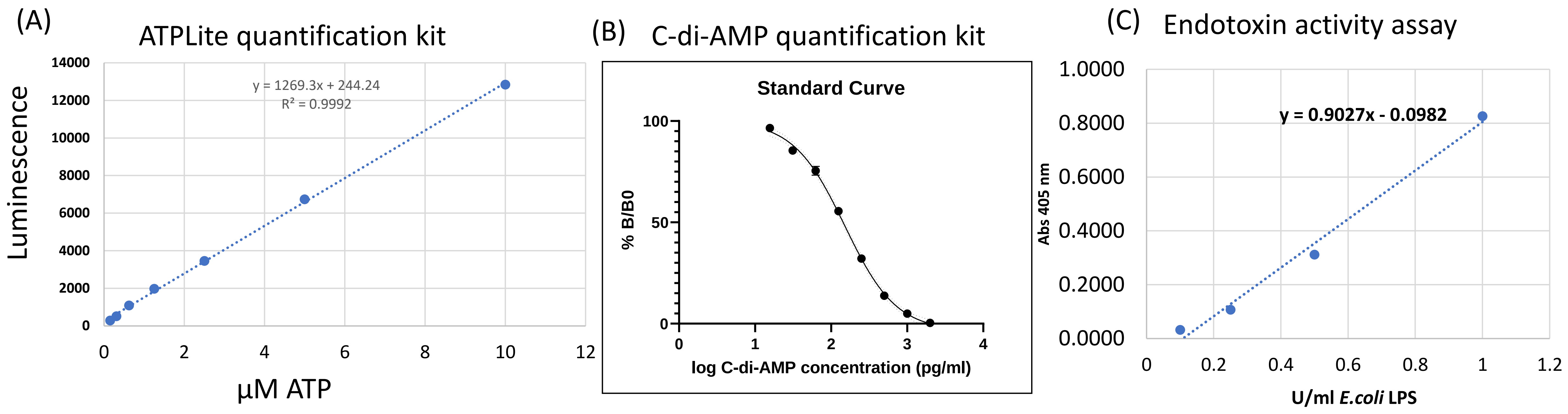

Fig. S11.
