# Supplementary material for: The multifaceted role of c-di-AMP signaling in the regulation of Porphyromonas gingivalis lipopolysaccharide structure and function
Source: Front Cell Infect Microbiol. 2024 Jun 12;14:1418651. doi: 10.3389/fcimb.2024.1418651 (PMC11199400; doi:10.3389/fcimb.2024.1418651)
Supplement: Supplementary file 2 [file Table_1.docx]

| **Table S1**. Retention time (RT) and intensities of different lipid classes in *P. gingivalis* WT and mutants, as well as pyruvate-treated condition | | | | | | | | | | |
| --- | --- | --- | --- | --- | --- | --- | --- | --- | --- | --- |
|  | RT | 6.35 | 8.21 | 8.14 | 6.06 | 6.1 | 7.67 | 6.58 | 6.58 | 1.81 |
|  | Sample | L654 | L1256 | L1242 | Unsub PGDHC | PEA | Sub PGDHC | PEDHC | L567 | L430 |
| Hemin 10 | ∆*pde_pg_* | 1214876 | 4723600 | 1838783 | 65346 | 5717124 | 2666300 | 4428453 | 34654 | 1559532 |
| Hemin 10 | ∆*cdaR* | 1599736 | 6336100 | 1612300 | 188050 | 5737734 | 3519000 | 4998500 | 5099 | 2478790 |
| Hemin 10 | WT | 1174239 | 4188105 | 1830600 | 13428 | 7064164 | 2029900 | 3094174 | 51968 | 2608426 |
| Hemin 1 | ∆*pde_pg_* | 1635957 | 5570100 | 1842835 | 80934 | 5268375 | 2900400 | 4755503 | 30644 | 1982140 |
| Hemin 1 | ∆*cdaR* | 1360779 | 4318278 | 1885819 | 11703 | 6740984 | 2056700 | 3441036 | 72954 | 2058187 |
| Hemin 1 | WT | 1714105 | 5450000 | 1732815 | 171160 | 5687925 | 3307600 | 5195900 | 12767 | 2234875 |
| Hemin 1 | Pyruvate | 1652478 | 5707900 | 1519988 | 245380 | 6004347 | 3779400 | 5835000 | 2534 | 1090974 |
