# Supplementary material for: The multifaceted role of c-di-AMP signaling in the regulation of Porphyromonas gingivalis lipopolysaccharide structure and function
Source: Front Cell Infect Microbiol. 2024 Jun 12;14:1418651. doi: 10.3389/fcimb.2024.1418651 (PMC11199400; doi:10.3389/fcimb.2024.1418651)
Supplement: Supplementary file 3 [file Table_2.docx]

| **Table S2.** Proposed compositions and structure interpretations for major lipid A ions in MALDI-TOF MS of *P. gingivalis* lipid A. These values served as a guideline for our interpretation | | | | | | |
| --- | --- | --- | --- | --- | --- | --- |
| Lipid A variants | Calc  [M+Na]^+^ | Calc  [M-H]^-^ | MW | Composition | Structure | Structure |
| **IV** | 1807.24 | 1782.23 | 1784.24 | P_2_, GlcN_2_, i-17:0(3-OH)_2_, 16:0(3-OH)_2_, 16:0 | Pentaacyl, P2 | 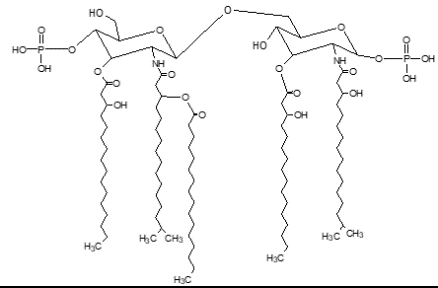 |
|  | 1793.35 | 1768.21 | 1770.35 | P_2_, GlcN_2_, i-17:0(3-OH)_2_, i-15:0(3-OH), 16:0(3-OH), 16:0 | Pentaacyl, P2 |  |
| **III** | 1725.26 | 1701.92 | 1702.26 | P, GlcN_2_, i-17:0(3-OH)_2_, 16:0(3-OH)_2_, 16:0 | Pentaacyl,  P1 | 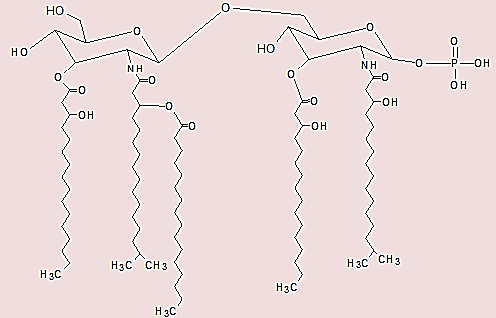 |
|  | 1711.25 | 1687.91 | 1688.25 | P, GlcN_2_, i-17:0(3-OH)_2_, i-15:0(3-OH), 16:0(3-OH), 16:0 | Pentaacyl,  P1 |  |
| **III** | 1647.42 | 1622.30 | 1624.42 | GlcN_2_, i-17:0(3-OH)_2_, 16:0(3-OH)_2_, 16:0 | Pentaacyl | 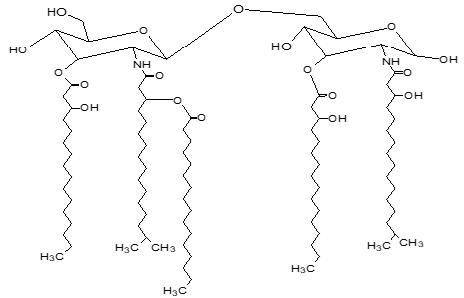 |
|  | 1633.39 | 1608.28 | 1610.39 | GlcN_2_, i-17:0(3-OH)_2_, i-15:0(3-OH), 16:0(3-OH), 16:0 | Pentaacyl |  |
| **II** | 1552.97 | 1528.01 | 1529.97 | P_2_, GlcN_2_, i-17:0(3-OH)_2_, 16:0(3-OH), 16:0 | Tetraacyl,  P2 | 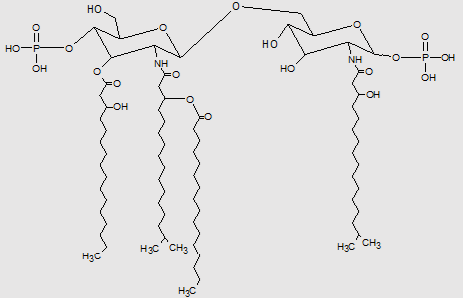 |
|  | 1538.94 | 1513.99 | 1515.94 | P_2_, GlcN_2_, i-17:0(3-OH)_2_, i-15:0(3-OH), 16:0 | Tetraacyl,  P2 |  |
|  | 1471.04 | 1447.62 | 1488.04 | P, GlcN_2_, i-17:0(3-OH)_2_, 16:0(3-OH), 16:0 | Tetraacyl,  P | 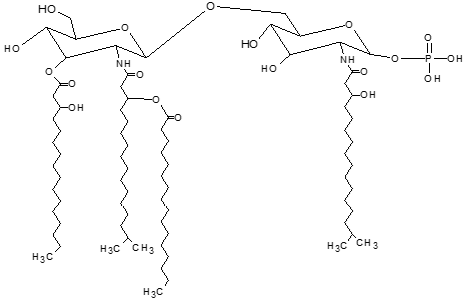 |
| **I** | 1457.02 | 1433.73 | 1434.02 | P, GlcN_2_, i-17:0(3-OH)_2_, i-15:0(3-OH), 16:0 | Tetraacyl,  P |  |
