# Supplementary material for: The multifaceted role of c-di-AMP signaling in the regulation of Porphyromonas gingivalis lipopolysaccharide structure and function
Source: Front Cell Infect Microbiol. 2024 Jun 12;14:1418651. doi: 10.3389/fcimb.2024.1418651 (PMC11199400; doi:10.3389/fcimb.2024.1418651)
Supplement: Supplementary file 4 [file Table_3.docx]

| **Table S3.** The major m/z signals observed in the negative reflector ionization mode in the MALDI-ToF MS analysis of lipid A isolated from *P. gingivalis* WT, Δ*pde_pg_* and Δ*cdaR* mutants | | | | | | | |
| --- | --- | --- | --- | --- | --- | --- | --- |
|  | Calc.  [M-H]^-^ | Observed m/z [M-H]^-^ | | | | | |
| Lipid A species |  | WT | Δ*pde_pg_* | Δ*cdaR* | WT | Δ*pde_pg_* | Δ*cdaR* |
|  |  | Hemin 1 | Hemin 1 | Hemin 1 | Hemin 10 | Hemin 10 | Hemin 10 |
| (pentaacyl bis-phosphoryl) | 1782.23 | ND | ND | ND | ND | ND | ND |
|  | 1768.21 | ND | ND | ND | ND | ND | ND |
| (pentaacyl mono-phosphoryl) | 1701.92 | 1702.01 | 1701.77 | 1701.79 | 1702.95 | 1701.93 | 1701.79 |
|  | 1687.91 | 1688.00 | 1687.76 | 1687.78 | 1687.94 | 1687.91 | 1687.81 |
| (pentaacyl) | 1622.30 | ND | ND | ND | ND | ND | ND |
|  | 1608.28 | ND | ND | ND | ND | ND | ND |
| (tetraacyl *bis*-phosphoryl) | 1528.01 | ND | ND | ND | ND | ND | ND |
|  | 1513.99 | ND | ND | ND | ND | ND | ND |
| (tetraacyl mono-phosphoryl) | 1447.62 | 1447.83 | 1447.62 | 1447.64 | 1447.77 | ND | 1447.66 |
|  | 1433.73 | 1433.82 | 1433.62 | 1433.64 | 1433.75 | 1433.73 | 1433.65 |
| (tetraacyl) | 1367.76 | 1367.84 | 1367.63 | 1367.66 | 1367.78 | 1367.76 | 1367.69 |
|  | 1354.06 | 1353.82 | ND | 1353.66 | ND | 1353.75 | 1353.68 |
