# Supplementary material for: The multifaceted role of c-di-AMP signaling in the regulation of Porphyromonas gingivalis lipopolysaccharide structure and function
Source: Front Cell Infect Microbiol. 2024 Jun 12;14:1418651. doi: 10.3389/fcimb.2024.1418651 (PMC11199400; doi:10.3389/fcimb.2024.1418651)
Supplement: Supplementary file 5 [file Table_4.docx]

| Table S4. The major m/z signals observed in the positive reflector ionization mode in the MALDI-ToF MS analysis of lipid A isolated from *P. gingivalis* WT, Δ*pde_pg_* and Δ*cdaR* mutants | | | | | | | |
| --- | --- | --- | --- | --- | --- | --- | --- |
|  |  | Observed Ions | | | | | |
| Lipid A species | Calc [M+Na]^+^ | WT | Δ*pde_pg_* | Δ*cdaR* | WT | Δ*pde_pg_* | Δ*cdaR* |
|  |  | Hemin 1 | Hemin 1 | Hemin 1 | Hemin 10 | Hemin 10 | Hemin 10 |
| (pentaacyl bis-phosphoryl) | 1807.24 | ND | ND | ND | ND | ND | ND |
|  | 1793.35 |  |  |  |  |  |  |
| (pentaacyl mono-phosphoryl) | 1725.26 | 1726.07 | 1726.13 | 1726.10 | ND | 1726.14 | ND |
|  | 1711.25 | 1712.06 | 1712.13 | 1712.10 | 1712.10 | 1712.14 | 1712.10 |
| (pentaacyl) | 1647.42 | 1646.08 | 1646.19 | ND | 1646.18 | 1646.19 | ND |
|  | 1633.39 | 1632.10 | 1632.18 | 1632.14 | 1632.17 | 1632.19 | 1634.11 |
| (tetraacyl *bis*-phosphoryl) | 1552.97 | ND | ND | ND | ND | ND | ND |
|  | 1538.94 | ND | ND | ND | ND | ND | ND |
| (tetraacyl mono-phosphoryl) | 1471.04 | ND | ND | ND | ND | ND | ND |
|  | 1457.02 | 1457.87 | ND | ND | ND | 1457.94 | ND |
| (tetraacyl) | 1391.07 | 1391.92 | ND | 1391.96 | 1391.98 | 1392.00 | 1391.96 |
|  | 1378.99 | 1377.91 | 1377.98 | 1377.96 | 1377.97 | 1377.99 | 1377.95 |
