# Supplementary material for: The multifaceted role of c-di-AMP signaling in the regulation of Porphyromonas gingivalis lipopolysaccharide structure and function
Source: Front Cell Infect Microbiol. 2024 Jun 12;14:1418651. doi: 10.3389/fcimb.2024.1418651 (PMC11199400; doi:10.3389/fcimb.2024.1418651)
Supplement: Supplementary file 6 [file Table_5.docx]

| **Table S5.** Absolute intensities of each band in gel mobility shift assays that were conducted under native electrophoresis conditions to investigate the binding potential of LPSs from WT and mutants to polymyxin B. | | | | | |
| --- | --- | --- | --- | --- | --- |
|  | LPS samples | No polymyxin B | | + polymyxin B | |
|  |  | Top band | bottom band | Top band | bottom band |
| Hemin 10 | ∆*pde_pg_* | 22871 | 25310 | 17858 | 7939 |
|  | ∆*cdaR* | 22561 | 25175 | 18870 | 8756 |
|  | WT | 16150 | 25256 | 7829 | 11006 |
| Hemin 1 | ∆*pde_pg_* | 13662 | 24734 | 8436 | 11824 |
|  | ∆*cdaR* | 20930 | 27494 | 17605 | 13692 |
|  | WT | 12888 | 26147 | 6342 | 11851 |
